# Structural organization of the spongy mesophyll

**DOI:** 10.1111/nph.17971

**Published:** 2022-02-15

**Authors:** Aleca M. Borsuk, Adam B. Roddy, Guillaume Théroux‐Rancourt, Craig R. Brodersen

**Affiliations:** ^1^ 5755 School of the Environment Yale University New Haven CT 06511 USA; ^2^ 5450 Department of Biological Sciences Institute of Environment Florida International University Miami FL 33199 USA; ^3^ Department of Integrative Biology and Biodiversity Research Institute of Botany University of Natural Resources and Life Sciences, Vienna 1180 Vienna Austria

**Keywords:** 3D, cellular organization, leaf anatomy, mesophyll, microCT, photosynthesis

## Abstract

- Many plant leaves have two layers of photosynthetic tissue: the palisade and spongy mesophyll. Whereas palisade mesophyll consists of tightly packed columnar cells, the structure of spongy mesophyll is not well characterized and often treated as a random assemblage of irregularly shaped cells.
- Using micro‐computed tomography imaging, topological analysis, and a comparative physiological framework, we examined the structure of the spongy mesophyll in 40 species from 30 genera with laminar leaves and reticulate venation.
- A spectrum of spongy mesophyll diversity encompassed two dominant phenotypes: first, an ordered, honeycomblike tissue structure that emerged from the spatial coordination of multilobed cells, conforming to the physical principles of Euler’s law; and second, a less‐ordered, isotropic network of cells. Phenotypic variation was associated with transitions in cell size, cell packing density, mesophyll surface‐area‐to‐volume ratio, vein density, and maximum photosynthetic rate.
- These results show that simple principles may govern the organization and scaling of the spongy mesophyll in many plants and demonstrate the presence of structural patterns associated with leaf function. This improved understanding of mesophyll anatomy provides new opportunities for spatially explicit analyses of leaf development, physiology, and biomechanics.

## Introduction

The laminar leaf with reticulate venation is common among terrestrial vascular plants, and this form has independently evolved in at least four lineages since the Paleozoic (Boyce & Knoll, 2002). Though there is large variation in leaf gross morphology (size, shape, and structure) (Wright *et al*., 2017), most laminar leaves have an interior mesophyll tissue that differentiates dorsiventrally during development, giving rise to two distinctly structured cell layers (Nicotra *et al*., 2011): the palisade and the spongy mesophyll. The palisade mesophyll is generally located below the upper epidermis and is composed of cylindrically shaped cells oriented perpendicular to the leaf surface. This layer is characterized by a high surface area to volume ratio that facilitates CO_2_ absorption in a region of the leaf where light is abundant and photosynthetic rates are high (Parkhurst & Mott, 1990; Ho *et al*., 2016; Théroux‐Rancourt *et al*., 2017, 2021; Borsuk & Brodersen, 2019). The spongy mesophyll, positioned below the palisade, is traditionally considered to be an irregular (Govaerts *et al*., 1996; Ivanova & P’yankov, 2002; Morison & Lawson, 2007; Terashima *et al*., 2011) or loosely packed (Nobel, 1999; Chatelet *et al*., 2013; Ho *et al*., 2016) assemblage of cells that are approximately spherical (Smith *et al*., 1997; Nobel, 1999) or of uncertain shape (Govaerts *et al*., 1996; Aalto & Juurola, 2002; Chatelet *et al*., 2013). Prior investigations focusing primarily on two‐dimensional (2D) transverse slices revealed no clear order to the spongy mesophyll or rules that define its structure (Govaerts *et al*., 1996). For simplicity, model assumptions derived from these descriptions commonly approximate the spongy mesophyll layer as a random arrangement of cells (Govaerts *et al*., 1996) with spherical or capsule geometry (Parkhurst, 1994; Govaerts *et al*., 1996; Aalto & Juurola, 2002; Chatelet *et al*., 2013). Although more spatially explicit examples of branching (Zhang *et al*., 2020), mesh‐like (Haberlandt, 1914; Wylie, 1931; Esau, 1965; Chandrasekharam, 1972), or foam‐like (Gibson *et al*., 1988; Niklas, 1992) tissue in the spongy mesophyll have been described, limited data exist on its structural organization across taxa, or how structural variation correlates with leaf function.

The spongy mesophyll must perform multiple biophysical functions (Haberlandt, 1914), such as scattering and absorbing light and promoting CO_2_ diffusion from the stomata to the palisade (Haberlandt, 1914; Smith *et al*., 1997; Terashima *et al*., 2011). However, testing how the structure of the spongy mesophyll influences leaf function requires detailed characterization that has been difficult to achieve due to the small scale and complex three‐dimensional (3D) arrangement of its cells and intercellular airspaces (IASs) (Earles *et al*., 2019). Unlike other plant tissues that are composed of confluent cells with little air space between them, a substantial fraction of the leaf mesophyll volume is the IAS between the mesophyll cells (e.g. up to 71% by volume; Earles *et al*., 2018). Because the surface area of the mesophyll cells exposed to the IAS is the absorptive surface through which CO_2_ diffuses for photosynthesis, variation in cell structure and arrangement can influence mesophyll conductance to CO_2_, which represents a major limitation on photosynthetic performance (Schindelin *et al*., 2012; Lehmeier *et al*., 2017; Gago *et al*., 2020; Théroux‐Rancourt *et al*., 2021). We therefore sought to characterize the structural organization of the spongy mesophyll for a diverse set of plants, focusing on species with laminar leaves and reticulate venation, traits that are predominant among vascular plants (Boyce & Knoll, 2002). We used X‐ray micro‐computed tomography (microCT) imaging, a technique that allows for high‐resolution visualization and quantification of tissue structures and cellular geometry in three dimensions (Théroux‐Rancourt *et al*., 2017; Earles *et al*., 2018, 2019), as opposed to 2D methods such as light microscopy, in which 3D structure must be inferred from 2D images. We then investigated the structural drivers of variation in spongy mesophyll organization and explored the relationships between mesophyll structural properties and functional performance.

## Materials and Methods

### Plant material

Mature, fully expanded leaves were selected from 40 species from 30 genera and 24 families spanning a wide diversity of extant vascular plants. These species include several congeneric pairs and six *Viburnum* species (Supporting Information Table S1). To facilitate comparison of mesophyll structure, this sampling included only species with laminar leaves and reticulate venation. Plants had been grown in glasshouses and arboreta. Healthy, well‐watered plants were selected, the petiole or stem was excised, and the leaves wrapped in wet paper towels and immediately put in plastic bags. They were then transported to the microCT facility and scanned within 36 h of collection.

### Micro‐computed tomography data acquisition and reconstruction

MicroCT data were obtained at the Advanced Light Sources (ALS) at the Lawrence Berkeley National Lab (Berkeley, CA, USA) and at the TOMCAT Tomography beamline, Swiss Light Source (SLS; Paul Scherrer Institute, Villigen, Switzerland). Samples were prepared before each scan (< 30 min) by excising a *c*. 1.5–2 mm wide and *c*. 15 mm long piece of leaf tissue near the leaf midpoint and offset 5–10 mm from the edge of the mid vein. The cut edges of the tissue samples were oriented parallel to the nearest second‐order vein, if possible, to capture areoles bounded by high vein orders (i.e. greater than or equal to second‐order veins, depending on the species). Tissue samples were enclosed between two pieces of polyimide tape to prevent desiccation while allowing high X‐ray transmittance during the scan. They were mounted in the sample holder, centered in the microCT X‐ray beam, and scanned using the continuous tomography mode, which captured 1025 (ALS) or 1800 (SLS) projection images at 21 keV, using a 5×, 10×, or 40× objective lens, yielding final pixel resolutions between 1.277 and 0.1625 µm. Each scan was completed in 5 min (SLS) or *c*. 15 min (ALS).

Image reconstruction methods followed those described by Théroux‐Rancourt *et al*. (2017). Image stacks were cropped to remove tissue that was dehydrated, damaged, or contained artifacts from the imaging or reconstruction steps. Image processing was applied equally among scans using the Fiji distribution of ImageJ software (Schindelin *et al*., 2012). Replication of methods on *n* = 3 samples for a representative species (*Rhododendron* sp.) indicated low intraspecific variance (Table S1). We used light microscopy and scanning electron microscopy to view different sections of the leaf (adjacent to the leaf apex, base, and margins) and found the same patterns as in the midsection used for microCT imaging (Fig. S1).

### Leaf traits

**Fig. 1:**
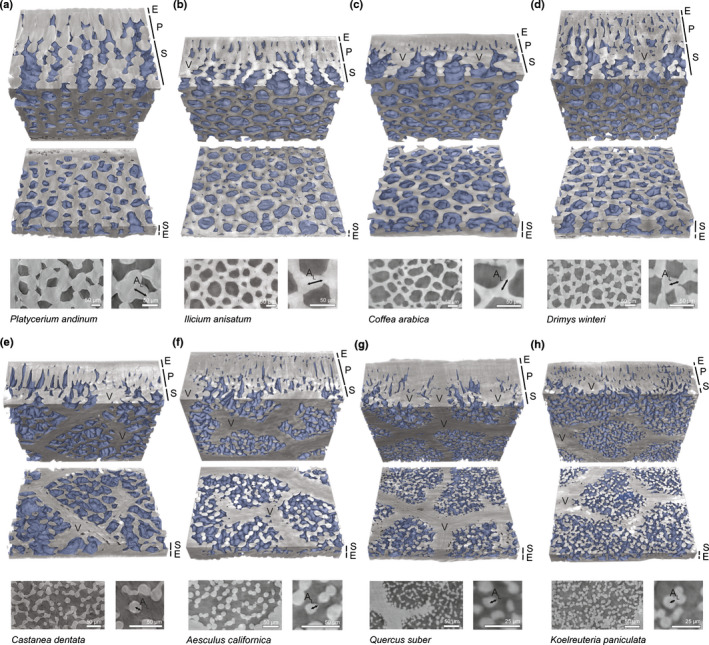
Representative views of spongy mesophyll and cell arm length measurements. Micro‐computed tomography volume renderings with paradermal bisections showing the epidermis (E), palisade mesophyll (P), spongy mesophyll (S), veins (V), and intercellular airspace (shaded blue) of representative species. Veins are seen in the paradermal plane only in (e–h) and form long, branching structures. Tissue dimensions for (a) *Platycerium andinum* are *c*. 1 mm across the horizontal edge; (b–h) tissue dimensions for all other species are *c*. 0.4 mm across the horizontal edge. Scale varies with perspective. Two‐dimensional slices are shown below the respective three‐dimensional rendering for each species at two magnifications to illustrate cell arm length *A*_L_ measurements. Cell arm length was measured by visual inspection from the tip of the arm to the common vertex of the arms. (e–h) Where cell arms were negligible or absent, *A*_L_ was analogous to cell radius. Species are shown from (a) to (h) in descending order of mean cell arm length; that is, *P. andinum* had the longest cell arms.

Most spongy mesophyll cells had multiple lobes or arms protruding from common vertices with a characteristic length dimension. Cell arm length *A*_L_ was therefore measured by visual inspection from the tip of the arm to the common vertex of two or more arms in the paradermal plane (Figs 1a–d, S2). Where cell arms were negligible or absent, *A*_L_ was analogous to cell radius (Fig. 1e–h). Spongy mesophyll cell arms were commonly tapered. For consistency, cell arm diameter *A*_D_ was measured at the *A*_L_ midpoint and, along with leaf thickness and spongy mesophyll thickness, was measured from microCT images of transverse leaf cross‐sections (Figs S1, S2). Spongy mesophyll *A*_L_, guard cell length, and stomatal width were measured from paradermal microCT slices. Mean and SE were calculated from replicates of 15–20 measurements of individual cells, with one arm measured per cell. Stomatal and cell packing density were measured from paradermal microCT images. Stomatal counts (*c*. 10–60 per sample) and spongy mesophyll cell counts (*c*. 40–140 per sample) were measured from areas between veins. All measurements were obtained using Fiji. To account for the spatial constraints on local mesophyll topology imposed by veins, we measured minimum vein spacing distance, defined as the shortest characteristic distance between veins and measured as the mean length between the highest order veins and closest neighboring veins (Fig. S3). Minimum vein spacing was measured from microCT image paradermal sections replicated 3–10 times per sample using Fiji. Measured minimum vein spacing data were complemented by published vein density data. Published data sources (Amiard *et al*., 2005; Feild & Balun, 2008; Boyce *et al*., 2009; Boyce, 2009; Brodribb & Feild, 2010; Walls, 2011; Sack *et al*., 2012; Sack & Scoffoni, 2013; Nardini *et al*., 2014; Scoffoni *et al*., 2016; Théroux‐Rancourt *et al*., 2021) are referenced in Table S1. Genome size data were taken from the Plant DNA C‐values database (release 7.1, 2019; Pellicer & Leitch, 2020).

#### Maximum photosynthetic rate

Rates of maximum photosynthesis *A*_max_, were obtained using empirical and literature data. For literature reported values, we used data stated as maximum photosynthetic rate or estimated the maximum photosynthetic rate from light response curves. In cases where no literature values were available, we measured maximum photosynthetic rates using an Li‐6400 portable gas‐exchange system (Licor Inc., Lincoln, NE, USA). Leaves were placed in the chamber and allowed to acclimate to the following conditions until they reached a steady state: photosynthetic photon flux density, 0, 50 100, 200, 400, 600, 800, 1000 µmol m^−2^ s^−1^; CO_2_, 400 ppm; relative humidity, 37%; vapor pressure deficit, 1.6 kPa; leaf temperature, 25°C. Measurements were performed on three or four leaves per species. Published data sources (Lu *et al*., 1997; Martindale & Leegood, 1997; Fernandez *et al*., 2002; Lusk & Del Pozo, 2002; Olsen *et al*., 2002; Takahashi *et al*., 2002; Chen & Cheng, 2003; Feild *et al*., 2003, 2009; McElrone & Forseth, 2004; Ronchi *et al*., 2006; Calatayud *et al*., 2007; Stewart *et al*., 2007; Brodersen *et al*., 2008; Vaz *et al*., 2010; Greer & Weedon, 2012; Ye *et al*., 2012; Chatelet *et al*., 2013; Fellows & Goulden, 2013; Kaiser *et al*., 2016; Martinez & Fridley, 2018) are referenced in Table S1.

#### Spongy mesophyll porosity and surface area

Image stacks were cropped to spongy mesophyll domains by excluding veins, palisade mesophyll, and epidermal layers, and the airspace was segmented by visually and subjectively defining a range of pixel intensity values that optimized airspace classification while minimizing false classification. The ImageJ plugin BoneJ2 (Doube *et al*., 2010; Domander *et al*., 2021) was then used to quantify spongy mesophyll IAS volume *V*_IAS_ (µm^3^), the total spongy mesophyll volume *V*_mes_ (µm^3^), and the spongy mesophyll surface area exposed to the IAS SA_mes_ (µm^2^). Mesophyll porosity (m^3^ m^−3^) was calculated as the IAS volume fraction of the total spongy mesophyll volume. Spongy mesophyll surface area per unit tissue volume was calculated as the ratio SA_mes_/*V*_mes_ (µm^2^ µm^−3^). Mesophyll surface area per projected leaf area *S*_m_ (m^2^ m^−2^) was calculated as the ratio SA_mes_/SA_leaf_ (m^2^ m^−2^), where SA_leaf_ (m^2^) is the width multiplied by the height of the leaf tissue in the transverse plane.

#### Pore network analysis

Simulation software (Avizo 2019.4; Thermo Scientific, Hillsoboro, OR, USA) was used to model the 3D connectivity and geometry of the IAS networks and to model material properties such as tortuosity and flow directionality. Image stacks cropped to isolated spongy mesophyll volumes were imported into the Avizo xporenetworkmodeling extension. IAS connectivity was measured by running the Volume Fraction module on each of the total airspace and interconnected airspace objects. The Generate Properties function was used to approximate vapor‐phase flow through the network using boundary values of 40 Pa and 25 Pa as input and output pressure (Sharkey *et al*., 1982), respectively, and a fluid viscosity of 1.837 × 10^–5^ Pa s. Tortuosity *τ* was defined as the fraction of the shortest pathway ∆*l* through the network and the Euclidean distance between the starting and end points of that pathway ∆, such that *τ =* ∆*l/*∆*x*. Tortuosity was evaluated in the vertical direction; that is, normal to the plane representing the evaporative leaf surface. For full details on the pore network analysis, see Methods S1.

#### Nearest neighbors

The nearest‐neighbor classification approach was used to find the number of edges (i.e. polygon class) of each spongy mesophyll IAS pore in the paradermal plane by first determining the center of each pore, then computing a Voronoi diagram, and subsequently counting the number of sides of the Voronoi cell associated with each pore. Nearest‐neighbor analysis was implemented using the ImageJ plugin Biovoxxel (Brocher, 2015). For images of hexagonal lattices cropped to rectangles with similar side lengths, the classification accuracy (percentage of polygons with six neighbors, i.e. ‘% 6N’) decreased sharply below *c*. 100 pores. Therefore, a minimum of *c*. 100 pores was included in each spongy mesophyll image for the nearest‐neighbor analysis to reduce edge effects (Fig. S4). For full details on the nearest‐neighbor analysis, see Methods S2.

#### Principal components analysis

Data for minimum vein spacing and flow rate directionality traits were log‐transformed to improve normality prior to analysis. Data for 17 leaf, tissue, and cell anatomy traits (Tables S1, S2) were standardized and evaluated by principal components analysis (PCA) using the prcomp function from the R package stats (v.3.6.23) in R (R Core Team, 2018). A scree plot (Fig. S5) was used to examine the percentage variation explained by each principal component (PC). The first two PCs explained 70.7% of variation in the data (58.3% and 12.4% for PC 1 and PC 2, respectively), with 7.6% of variation explained by the third PC. The first two PCs were therefore retained, with interpretation of the data based primarily on PC 1 (Fig. S5). Eigenvector scores showing the associations between PCs and traits are provided in Table S2.

#### Cluster analysis

Using the same dataset analyzed by PCA (Table S2), hierarchical cluster analysis was used to group species according to similar traits, or proximity, in multidimensional space (Fig. S6). Proximity was measured using Euclidean distance, and clusters were agglomerated using Ward’s linkage. Analysis was performed using the hclust function using the ward.D2 method from the R package stats in R. Clusters were pruned using the rect.hclust function in R.

#### Phenotype assignment

Samples with a contiguous lattice structure in the paradermal plane of the spongy mesophyll were assigned a honeycomblike phenotype. Assignments were cross‐validated in three dimensions with IAS network visualizations and network flow rate analysis (Table S1). Apart from *Gnetum gnemon*, all species with the honeycomb spongy mesophyll exhibited a vertical to lateral flow rate ratio ≥ 3.8. This approximate threshold in the functional properties of the tissue reflects both the size and orientation of the IAS channels and indicates the presence of prominent vertical IAS channels characteristic of a 3D honeycomb. It is possible the fibers situated close to the abaxial and adaxial epidermis in *G*. *gnemon* disrupt the registration of lattice layers, and thus the organization of the honeycomb in 3D; yet, because the 2D lattice layers were robust, *G*. *gnemon* was considered to have the honeycomb phenotype.

#### Intercellular airspace pore diameter and count

The 2D IAS pore diameter and count were measured for lattice characterization from paradermal microCT images processed following the same method as in the nearest‐neighbor analysis. Pore area and count were measured using the Particle Analyzer tool in ImageJ. Pore diameter *D*_pore_ was calculated in R from pore area *A*_pore_ assuming a circular geometry:

(Eqn 1) $$D_{\text{pore}}=\sqrt{\frac{4A_{\text{pore}}}{\pi }}$$

Mean and SD were then calculated for each species (Table S1).

#### Euler characteristics *θ*, *Z*_e_, and $\bar{n}$

The topological constraints of Euler’s law (Gibson & Ashby, 1999) determine the number of vertices *V*, edges *E*, and faces (IAS voids *F*) in a large 2D aggregate according to:

(Eqn 2) F‐E+V=1

so that in a lattice with six edges *E* surrounding each face *F* the number of vertices *V* per face will also be six. This also results in an edge connectivity *Z*_e_ of three, resulting in a hexagonal honeycomb that tessellates 2D space with the least material investment (Hales, 2001). To validate the applicability of Euler’s law for 2D paradermal slices of spongy mesophyll, we first measured the characteristic internal angle *θ* using BoneJ2 (Doube *et al*., 2010; Domander *et al*., 2021). The ‘Use clusters’ pruning method simplified densely clustered nodes. Edge connectivity *Z*_e_ ranged from 3 to 10. We calculated inter‐edge angle and edge connectivity for each species, and mean and SD were then calculated for the dataset (*n* = 29). The number of edges per face $\bar{n}$ was calculated from the edge coordination relationship:

(Eqn 3) $$\bar{n}=\frac{2Z_{e}}{Z_{e}‐2}$$

which is a generalized application of Euler’s law (Eqn 2) for a nonregular honeycomb, or for a 2D aggregate with varying polygon classes. A Welch two‐sample *t*‐test was done to test agreement between $\bar{n}$ and the nearest‐neighbor measurements. A two‐sided analysis (*t*(55) = 11.2, *P* < 0.001) showed there was no significant difference between $\bar{n}$ (mean 5.89, SD 0.07) and nearest neighbors (mean 5.69, SD 0.06).

#### Tessellation entropy, Lewis’ rule, and Aboav–Weaire law

Topological methods can be used to describe how ordered or regular a structure is. We described dispersion in the IAS pore polygon class of the spongy mesophyll using tessellation entropy and by comparison with theoretical predictions from Lewis’ rule and the Aboav–Weaire law. Lewis’ rule of polygon size dispersion is derived from the space‐filling properties of cucumber epithelial cells (Lewis, 1928, 1931) and indicates the degree of uniformity within a lattice by relating the area of the individual polygon classes $A_{n}$ to the mean $A_{\bar{n}}$. For highly uniform lattices such as cucumber epithelia (Lewis, 1931), the range of the distribution of areas for each polygon class is restricted around the mean, and the relationship between polygon class and the Lewis ratio $A_{n}/A_{\bar{n}}$ is linear. In coarse lattices (Lewis, 1931), the average areas of the polygon classes are more divergent, and the relationship between polygon class and the Lewis Ratio is nonlinear. An additional property of lattice order is given by the Aboav–Weaire law (Weaire, 1974), where the occurrence of a polygon with a lower than average number of edges (*n* < 6) introduces a corresponding polygon with a higher number of edges into the aggregate, frequently as a neighbor. For full details on the theory and methods of tessellation entropy, Lewis’ rule, and the Aboav–Weaire law, see Methods S3.

#### Random forest classification

Random forest classification was implemented using the randomforest package (Liaw & Wiener, 2002) in R using an expanded suite of continuous and categorical predictors to cross‐validate the PCA and cluster analyses and to identify the specific range of values for each predictor trait where phenotypes diverge. An ensemble of 5000 decision trees with four variables per node was examined, well exceeding the point at which out‐of‐bag (OOB), honeycomb, and nonhoneycomb error rate stabilized (*c*. 2200 trees). OOB error estimate was 2.5%; that is, 97.5% of samples were correctly classified. The confusion matrix (Table S3) further shows a class error of 0% for the honeycomb phenotype and a class error of 0.09% for nonhoneycomb mesophyll. Partial dependence plots (Liaw & Wiener, 2002) show the marginal effect of a variable on the relative likelihood of classification. The variable importance plot (Liaw & Wiener, 2002) indicates how important each variable is in classifying the data, with the most important variables at the top of the plot. Importance of variables is measured using mean decrease in accuracy.

### Power law and linear regression modeling

Allometric relationships were calculated from log‐transformed data via standardized major axis regression using the package smatr (Warton *et al*., 2012) in R, and linear relationships were calculated from normally distributed, nontransformed data using the function lm in R.

To determine the evolutionary coordination between traits, we constructed a phylogeny from the list of taxa using Phylomatic (v.3) and its stored family‐level supertree (v. R20120829) using the R package brranching (Chamberlain, 2018). Following published methods (Simonin & Roddy, 2018), we compiled node ages of named crown groups from fossil‐calibrated estimates of crown group ages (Lu *et al*., 2014; Magallón *et al*., 2015; Testo & Sundue, 2016). Of the 32 internal nodes in our phylogeny, 31 of them had published ages, which were assigned to nodes and branch lengths between dated nodes smoothed using the function bladj in the software Phylocom (v.4.2) (Webb *et al*., 2008). We tested whether there was correlated evolution between traits (Table S4) using phylogenetic least‐squares regression with a Brownian motion correlation structure using the R packages nlme (Pinheiro *et al*., 2018) and ape (Paradis *et al*., 2004). Traits were log‐transformed to improve normality prior to regression analyses.

### Surface area and volume calculations of idealized isodiametric and triply lobed cells

Surface areas of isodiametric and triply lobed cells with the same volume were approximated using idealized 3D geometrical representations. Isodiametric cells were modeled as spheres (Fig. S7a), and triply lobed cells were modeled as three cylinders connected to a triangular prism with an equilateral base (Fig. S7b). For full details on modeling of isodiametric vs triply lobed cells, see Methods S4.

## Results

Cell arm length (Fig. 1) and diameter covaried and were best characterized by a power law relationship (*R* ^2^ = 0.81, *P* < 0.001; Fig. S8; Table S5). There were two unoccupied regions of the cell geometry trait space. First, no species in our dataset had large, isodiametric cells. Such a cell would have an extremely large volume, as found in succulent and epiphytic plants whose leaves are often not laminar (Nelson & Sage, 2007; Earles *et al*., 2018). Second, there were no species that had cells with highly elongated, narrow arms (i.e. *A*_L_ > 54 µm and *A*_D_ < 6 µm; Table S1). Cell arm length was significantly correlated (Pearson correlation *r*_p_) with other leaf traits (Table S5), such that increases in cell arm length reflected increases in the characteristic dimensions of the entire structure; for example, cell arm diameter (*r*_p_ = 0.89, *P* < 0.001), stomatal guard cell length (*r*_p_ = 0.78, *P* < 0.001), and the diameter of the IAS pores (*r*_p_ = 0.90, *P* < 0.001). These relationships remained significant after accounting for shared evolutionary history (Table S4).

**Fig. 2:**
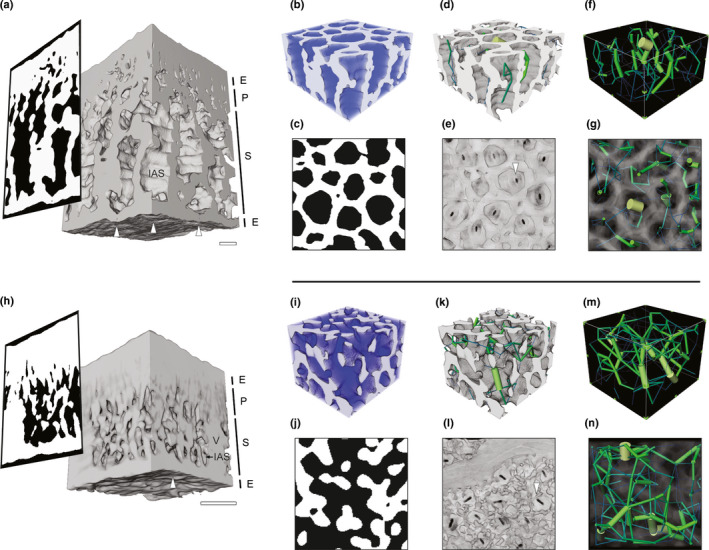
Spongy mesophyll tissue and intercellular airspace (IAS) network structure. (a) Representative leaf with relatively larger IAS conduit radii in the vertical vs lateral directions (*Berberis nervosa*). Two‐dimensional (2D) transverse view with cells shown in white and IAS in black. Three‐dimensional view of epidermis (E), palisade mesophyll (P), spongy mesophyll (S), and vascular tissue (V) shown in grayscale. The positions of representative stomata are indicated with white arrows. Bar, 50 µm. (b) Connectivity map of honeycomb spongy mesophyll. Blue areas indicate IAS regions connected to the network. Cells shown in white. (c) 2D paradermal view of honeycomb spongy mesophyll with cells shown in white and IAS in black. (d) IAS network schematic with tissue; cylinders scaled (50%) in size by IAS conduit cross‐sectional area and colored by IAS conduit radius (range *c*. 0.3 µm (dark blue) to 30 µm (yellow)) and tissue (grayscale). (e) Paradermal view of honeycomb spongy mesophyll IAS conduits and abaxial stomata. White arrow indicates position of representative stomate. (f) IAS network view (same as in (d) without tissue). (g) Paradermal view of IAS network and tissue structure (semitransparent). (h–n) Representative leaf with similar IAS conduit radii in the vertical and lateral directions (*Quercus suber*); panels follow the same order as (a–g). IAS network cylinders in (k), (m), and (n) scaled (50%) in size by IAS conduit cross‐sectional area and colored by IAS conduit radius (range *c*. 0.3 µm (dark blue) to 13 µm (yellow)).

Although the IAS frequently appeared segmented into discrete domains bounded by cells in 2D paradermal and transverse planes of the leaf (Fig. 2a,h), 3D network analysis indicated the IAS was fully interconnected in all species (median fraction of interconnected IAS 99.99%; interquartile range (IQR) 99.44–99.98%). Because cells must be interconnected to be metabolically viable, our IAS analysis supports a characterization of the spongy mesophyll as bicontinuous, meaning that the interwoven IAS and cellular networks are self‐continuous pathways (Scriven, 1976). The prevailing vertical orientation of relatively large IAS channels promoted highly directional, or anisotropic, modeled flux of CO_2_ in many species, such as *Berberis nervosa* (vertical : lateral flow rate ratio of 90.1, IAS channel geometry shown in Fig. 2d–g). However, IAS structure varied widely across our dataset, with species such as *Quercus suber* showing relatively less directionally biased, or more isotropic, IAS channels and, therefore, transport properties (vertical : lateral flow rate ratio of 3.6, IAS channel geometry shown in Fig. 2k–n). Mesophyll porosity, a commonly used metric, provided minimal insight into the observed differences in IAS structure and was not significantly related to functional traits such as maximum photosynthetic rate *A*_max_ (*R* ^2^ = 0.008, *P* = 0.64). For example, the spongy mesophyll of the two species shown in Fig. 2 had similar porosities (Table S1, 0.4705 and 0.5195 for *B. nervosa* and *Q*. *suber*, respectively) yet dissimilar IAS organizations.

### Multidimensional exploration of spongy mesophyll traits

**Fig. 3:**
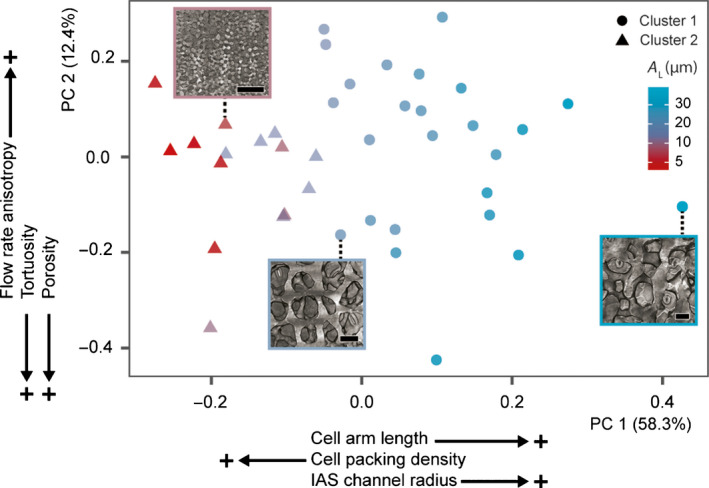
Patterns of variation in 17 spongy mesophyll and leaf traits across 40 species. Principal components (PCs) analysis ordination of species with major groups determined by cluster analysis shown by point shape. PC axes 1 and 2 explain 58.3% and 12.4% of the variance, respectively. Labels adjacent to the PC axes display traits with the highest eigenvector scores; labels with the highest scores are shown nearest to the axes. Eigenvector scores of all traits can be found in Supporting Information Table S2. Locations of individual species on the ordination plane are indicated by points and colored by mean cell arm length *A*_L_, the trait with the highest overall eigenvector score. Insets show micro‐computed tomography images of the spongy mesophyll (paradermal view) to illustrate the anatomical traits associated with various locations on the ordination plane and between clusters (from left to right: *Helianthus annuus*, *Calycanthus occidentalis*, *Platycerium andinum*. Bar, 50 µm). Major cluster analysis groups indicated by circular (cluster 1) and triangular (cluster 2) points. Cluster analysis relationships indicate the Euclidean distance between species in 17‐dimensional space by Ward’s agglomeration, where species within the same cluster show patterns of similarity.

To assess trends in spongy mesophyll traits, we used two multivariate statistical methods to explore leaf, tissue, and cell anatomical traits (Tables S1, S2). First, we reduced the 17 trait dimensions using PCA (Figs 3, S5). The first PC axis, accounting for 58.3% of the variance, had the highest eigenvector scores for anatomical factors such as mean *A*_L_, cell packing density, and IAS channel radius (Table S2). PC axis 2, accounting for 12.4% of the variance, had the highest eigenvector scores for traits such as porosity, tortuosity, and flow rate directionality (Table S2).

We then used a cluster analysis to group species based on their proximity in 17‐dimensional trait space (Fig. S6; Table S1). The primary, or basal, cluster relationships were comparable to species locations along the first PCA axis, where species with longer cell arms were associated with a large cluster (cluster 1; circles in Fig. 3), and a subset of species with small cell arms were associated with a smaller cluster (cluster 2; triangles in Fig. 3). Towards the tips of the dendrogram, species were grouped closely by genus, such as those in the genera *Vitis*, *Quercus*, *Illicium*, and some members of *Viburnum* (Fig. S6). This clustering suggests that spongy mesophyll structural traits are conserved within genera. Together, the cluster analysis and the PCA suggest that spongy mesophyll may be grouped approximately into two regions of structural trait space and that these groupings are determined predominantly by traits such as cell arm dimensions, IAS pore dimensions, and cell packing density.

**Fig. 4:**
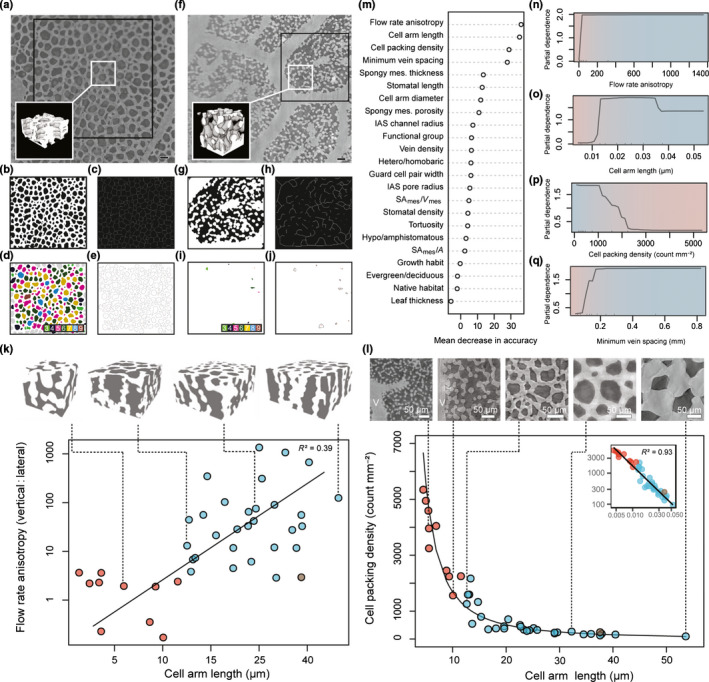
Phenotype assignment and random forest analysis of spongy mesophyll organization. Image processing and lattice measurements for (a–e) honeycomblike (*Illicium anisatum* shown) and (f–j) nonhoneycomb (no lattice; *Aesculus californica* shown) species. (a) Paradermal grayscale image taken with micro‐computed tomography. Bar, 50 µm. Inset: three‐dimensional (3D) rendering of tissue showing vertical stacking of cells. (b) Binary thresholded image. (c) Tissue skeleton image. (d) Intercellular airspace (IAS) pore nearest‐neighbor image; gray cells not included to minimize edge effects. (e) IAS pore outlines. (f–j) Analogous images for a representative nonhoneycomb species showing the absence of lattice properties. Phenotype assignments are approximate and do not preclude intermediate forms. (k) Scaling relationship (solid line) between cell arm length *A*_L_ and flow rate directionality (axes are log transformed). Species with honeycomb and nonhoneycomb spongy mesophyll are shown with blue and red circles, respectively. *Spinacia oleracea*, which was treated as nonhoneycomb in the analysis yet had a unique phenotype, is indicated with a brown circle. The 3D renderings above the horizontal axis show spongy mesophyll (white) and IAS (gray) for (from left to right) *Vitis vinifera*, *Ficus microcarpa*, *Nepenthes ventricosa*, and *Platycerium andinum*. (l) Power law relationship (solid line) between *A*_L_ and cell packing density. Images above the horizontal axis show spongy mesophyll for (from left to right) *Quercus kelloggi*, *Castanea dentata*, *F*. *microcarpa*, *Illicium anisatum*, and *P. andinum*. Figure inset shows the log–log transformed data and linear fit (solid line). Color scheme is same as in (k). (m) Variable importance plot for random forest classification between honeycomb and nonhoneycomb phenotypes. Predictors at the top of the ranking have a higher relative importance in classification, as determined by mean decrease in accuracy when these variables are removed from the model. (n–q) Partial dependence plots showing the natural logarithm of the odds of classification over the value range for the four traits with the highest relative importance. Blue shading shows approximate values over which probability favors the honeycomb phenotype; red shading shows approximate values over which probability favors the nonhoneycomb phenotype.

As supported by PCA and cluster analysis, two divergent spongy mesophyll phenotypes were observed using the microCT images and by comparison of IAS network properties (Fig. 4). Assignment of spongy mesophyll phenotype was based on the presence or absence of a honeycomblike lattice topology in the paradermal plane (Fig. 4a–j), an emergent property that arose from interactions between traits – such as A_L_, cell packing density, and IAS channel radius – that differentiated species on PC axis 1 (Nelson *et al*., 2005). In lattice‐forming, or honeycomblike, species, contiguous lattices of cells (Fig. 4a–c) enclosed IAS pores (29/40 species; Figs 1a–d, 4d,e; Table S1; Videos 1, 2). The spongy mesophyll of these species was tessellated by prismatic vertical air channels (Fig. 4a) typically positioned above stomata (Fig. 2a,e), forming the substomatal cavity and pathways that linked stomata in the lower leaf to the palisade layer (Figs 2d, S1c–f; Videos 1, 2). Using 3D IAS network analysis (Fig. 2), we found the columnar IAS channels were associated with more directionally biased, or anisotropic, CO_2_ flux (median vertical : lateral flow rate 33.2, IQR 11.8–90.1; Table S1) compared with species with nonlattice, or nonhoneycomb, tissue (median vertical : lateral flow rate 2.2, IQR 1.1–2.7). In 11/40 species dominated by the Rosid group of eudicots, no prismatic channels or paradermal cell lattices were observed (nonhoneycomb species; Figs 1e–h, 4f–j; Table S1; Videos 3, 4). Rather, lateral connectivity of airspace resulted in a more isotropic, or less directionally biased, network of air channels. These structures were consistent with prior descriptions of the spongy mesophyll as irregular (Govaerts *et al*., 1996; Ivanova & P’yankov, 2002; Morison & Lawson, 2007; Terashima *et al*., 2011).

Phenotypic assignments (honeycomb/nonhoneycomb) were approximations of dominant patterns in the spectrum of structural organization and do not preclude intermediate forms. Several species had transitional characteristics, for example five species placed into cluster 2 (predominantly nonhoneycomb) by cluster analysis were instead observed to have the honeycomblike phenotype (Fig. S5; *Ficus microcarpa*, *Aristolochia trilobata*, *Parthenocissus quinquefolia*, *Acer monspessulanum*, and *Viburnum utile*). These species had relatively small spongy mesophyll cell dimensions and high cell packing densities, yet they exhibited tissue lattices (Fig. S9) with vertically dominated IAS pathways (median vertical : lateral flow rate 7.4, IQR 6.8–13.1) compared with the nonhoneycomb species (median vertical : lateral flow rate 2.2, IQR 1.1–2.7). Deviation from the honeycomb and nonhoneycomb structures occurred within and outside of our dataset, including spinach (*Spinacia oleracea*, Fig. S10a), which had elongated cells radiating in all directions, and the aquatic water lily, *Nuphar polysepela*, which had laterally oriented IAS chambers presumably to increase airspace volume for buoyancy (Fig. S10b). Distinctive structures were observed in leaves with parallel venation (*n* = 4), in which chains of elongated cells attached to veins at right angles, forming an approximately rectilinear lattice (Fig. S11).

To explore what additional factors were involved in driving the structure of the spongy mesophyll, we used a random forest analysis to rank the importance of 23 anatomical, environmental, and taxonomic traits (Figs 4m, S12; Table S1) in accurately placing species into the observed honeycomb or nonhoneycomb phenotypes. The accuracy of random forest sorting of spongy mesophyll structure was most dependent on four traits: flow rate directionality, *A*_L_, cell packing density, and the characteristic minimum vein spacing (Fig. 4m). These findings are in general agreement with PCA determination of drivers in spongy mesophyll structural space, where *A*_L_ and cell packing density had the highest eigenvector scores on PCA axis 1 and flow rate directionality had the third highest eigenvector score on PCA axis 2. The results of the random forest analysis also elucidated steep transitional thresholds from honeycomb to nonhoneycomb phenotypes in flow rate directionality (Fig. 4n), *A*_L_ (Fig. 4o), cell packing density (Fig. 4p), and as minimum vein spacing fell below *c*. 0.1 mm (Fig. 4q). The transition between phenotypes also corresponded to notable thresholds (Fig. S12) in vein density and stomatal density. The nonhoneycomb phenotype was associated with vein density above *c*. 9 mm mm^−2^ and stomatal density above *c*. 260 mm^−2^. Therefore, leaves with smaller and more densely packed cells, isotropic IAS channels, and closely spaced veins were more likely to exhibit the nonhoneycomb topology, whereas leaves with larger and less densely packed cells, anisotropic IAS channels, and more distantly spaced veins were more likely to exhibit the honeycomb topology. The power law scaling found between spongy mesophyll cell arm length and traits such as cell packing density (*R* ^2^ = 0.93, *P* < 0.001; Fig. 4l; Table S6), flow rate directionality (*R* ^2^ = 0.39, *P* < 0.001; Fig. 4k; Table S6), and minimum vein spacing (*R* ^2^ = 0.65, *P* < 0.001; Table S6) indicates that nonlinear relationships dominate the spongy mesophyll structural trait space, with anatomical patterns that shift rapidly over certain thresholds.

### Honeycomb topology of the spongy mesophyll

**Fig. 5:**
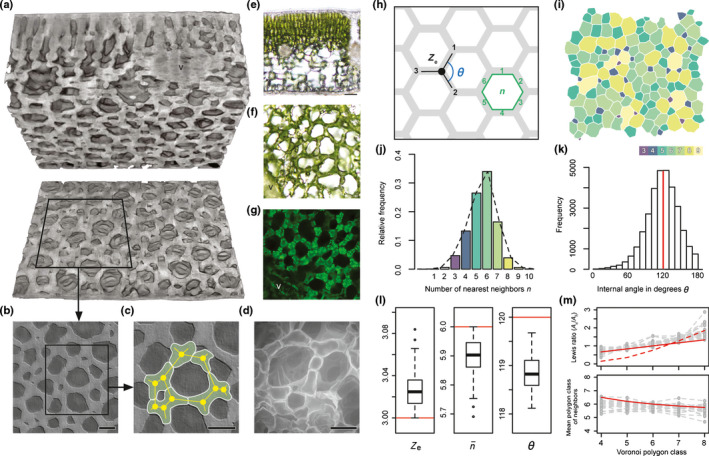
Variable cell shape, lattice emergence, and lattice characterization. (a) Leaf‐scale view of the spongy mesophyll in a representative species with the honeycomb phenotype (*Illicium anisatum* shown in (a–c); Bar, 50 µm). The leaf is sectioned in the paradermal plane near the abaxial surface. (b) Paradermal micro‐computed tomography image of spongy mesophyll layer. (c) Magnified view of spongy mesophyll construction. Individual cells highlighted with white borders. Yellow dots represent lattice vertices, and yellow lines represent lattice edges. Cell with three lobes shown in red; cells with more than three lobes shown in green. (d) Paradermal scanning electron microscope image of spongy mesophyll layer. (e) Representative transverse view of leaf with honeycomb spongy mesophyll ((e–g) show *Rhododendron* sp.) using brightfield microscopy. (f) Paradermal section with spongy mesophyll between veins. (g) Fluorescence microscopy showing spongy mesophyll cell walls (dark lines), chloroplasts (green points), and vascular tissue (v). (h) Schematic of lattice properties. Spongy mesophyll cell arms form edges (gray) that enclose intercellular airspace polygons (white) with internal angles *θ*. Mean edge connectivity is given by *Z*_e_, and edges per polygon is given by *n*. (i) Nearest‐neighbor diagram for a representative sample. (j) Frequency distribution of nearest neighbors for *n* = 29 species with the honeycomb phenotype. (k) Frequency distribution of *θ* for *n* = 29 species with the honeycomb phenotype. (l) Box plots for *Z*_e_, mean edges per face $\bar{n}$, and mean internal angle *θ* for *n* = 29 species with the honeycomb phenotype. Boxes represent interquartile range, lines across boxes represent group median, and whiskers extend from the upper and lower quartiles to the group maximum and minimum, respectively. Asterisks represent sample outliers. Red bars indicate values for a perfectly regular hexagonal honeycomb. (m) Comparison of predicted (red) and measured (gray) values for dispersion in polygon size and class for Lewis’ rule (upper panel, irregular lattice structure for an artificial emulsion given by the dotted red line) and the Aboav–Weaire law (lower panel).

The properties of the lattice‐forming tissue, including the presence of close‐packed prismatic IAS columns and highly directional flow, are properties of the honeycomb class of 3D cellular solids (Gibson & Ashby, 1999). Beyond the well‐known hexagonal structures made by bees, 3D honeycombs are generalized as close‐packed arrays of regular or irregular prisms (Gibson & Ashby, 1999). The honeycomblike topology of the spongy mesophyll emerged from local variation in cell shape and size, where cell arms joined together to form lattice edges (Fig. 5a–h). Though the honeycomb pattern was largely invariant according to standard topological indices (Fig. 5h–m; Table S1), the cells in each tissue sample had individually variable morphologies and arrangements (Fig. 5b,c). Cell morphology was validated for several species with brightfield (Fig. 5e,f), fluorescence (Fig. 5g), and environmental scanning electron microscopy (Fig. 5d). Tissue organization obeyed the topological constraints of Euler’s law (Fig. 5h). The most efficient polygonal lattices are hexagonal, which have internal angles *θ* of 120° (Fig. 5h) and minimize investment in materials needed to tessellate a 2D plane (Hales, 2001). Here, spongy mesophyll cells formed the lattice edges and vertices, and the IAS voids represented the internal polygons. The spongy mesophyll lattices were dominated by hexagons (Fig. 5i–k), with vertices joined by a mean edge connectivity of *Z*_e_ = 3.03 ± 0.02, a mean IAS number of edges of $\bar{n}=5.89\pm 0.07$, and a characteristic angle *θ* = 118.86 ± 0.40° (Fig. 5l; Table S1). To quantify the degree of order in the structure, we calculated the tessellation entropy *S* for each sample, which would be zero for a perfectly regular structure. We found a mean tessellation entropy of 1.43 ± 0.03, which is similar to values reported for engineered thin films with honeycomb morphologies dominated by hexagons (*S* = 1.48; Pietsch *et al*., 2009). The honeycomb structures also meet the assumptions for relatively uniform lattices as given by the Lewis rule and Aboave–Weaire law (Fig. 5m) and are comparable to well‐ordered biological structures such as cucumber epithelia (Lewis, 1931).

### Functional implications of spongy mesophyll structure

**Fig. 6:**
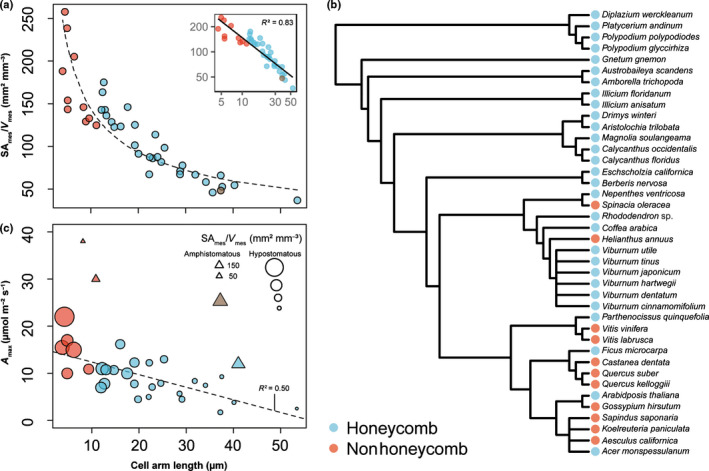
Spongy mesophyll cell arm length *A*_L_ and tissue structure in relation to leaf photosynthetic properties and phylogeny. (a) Relationship between spongy mesophyll surface‐area‐to‐volume ratio SA_mes_/*V*_mes_ and *A*_L_. Power law regression shown by the black dashed line. Colors represent spongy mesophyll structural pattern (red, nonhoneycomb; blue, honeycomb; brown, neither (*Spinacia oleracea*)). (b) Distribution of spongy mesophyll structural patterns among 40 land plant species with laminar, reticulately veined leaves. (c) Linear relationship between *A*_max_ and mean *A*_L_. Open circles represent hypostomatous species. Open triangles represent amphistomatous species, which were excluded from the regression. Point size scaled according to SA_mes_/*V*_mes_, which is linearly related to *A*_max_ (*R* ^2^ = 0.62).

To explore if spongy mesophyll structural traits predicted photosynthetic properties of the leaf, we examined the relationship between the spongy mesophyll cell surface area per unit tissue volume (SA_mes_/*V*_mes_) and *A*_L_. We found that, as arm length increased, SA_mes_/*V*_mes_ of the spongy mesophyll decreased sharply according to a power law (*R* ^2^ = 0.83, *P* < 0.001; Fig. 6a; Table S6). Thus, plants with larger cells, such as the honeycomb‐patterned fern *Platycerium andinum* (shown in Fig. 1a), had lower SA_mes_/*V*_mes_ than eudicots (Fig. 6b), such as *Helianthus annuus* (sunflower; shown in Figs 3a, S4, S5 insets) with smaller cells and the nonhoneycomb phenotype. Although cell size was strongly correlated with SA_mes_/*V*_mes_ (Théroux‐Rancourt *et al*., 2021), variation in cell geometry may also influence this property (Ivanova & P’yankov, 2002; Harwood *et al*., 2020). Using idealized models of isodiametric and triply armed cells (Fig. S7), we found surface area increased in triply armed cells compared with isodiametric cells of equal volume, with a mean difference in SA/*V* between the two modeled cellular geometries of 0.069 µm^2^ µm^−3^ (SD = 0.029 µm^2^ µm^−3^). Thus, in addition to cell size and cell packing density (Théroux‐Rancourt *et al*., 2021), cell shape can regulate SA_mes_/*V*_mes_.

Given that spongy mesophyll structure and surface area provide the physical basis for the conductance of CO_2_ for photosynthesis, we tested how leaf‐level maximum photosynthetic rate *A*_max_ was related to *A*_L_ and SA_mes_/*V*_mes_ (Fig. 6c). We found *A*_max_ decreased linearly with increasing *A*_L_ (*R* ^2^ = 0.50, *F*(1,27) = 26.65, *P* < 0.001). Higher *A*_max_ values occurred among species with nonhoneycomb phenotype (Table S1), whereas lower photosynthetic rate was associated with the honeycomb phenotype. Species with amphistomatous leaves, such as *H. annuus*, were excluded from the relationship (shown in in Fig. 6c as triangles), as the capacity for gas exchange on both sides of the leaf promotes higher photosynthetic rates (Muir, 2019). *A*_max_ increased linearly with increasing spongy mesophyll SA_mes_/*V*_mes_ (*R* ^2^ = 0.63, *F*(1,27) = 46.06, *P* < 0.001); thus, although the palisade mesophyll is typically modeled with a higher photosynthetic capacity relative to the spongy mesophyll (Ho *et al*., 2016), there is a strong positive relationship between the quantity of photosynthetically active SA_mes_/*V*_mes_ in the spongy mesophyll and leaf‐level photosynthetic capacity.

## Discussion

Given the historical dominance of 2D transverse analysis of leaves, our data highlight the importance of 3D characterization of mesophyll structure (Théroux‐Rancourt *et al*., 2017; Harwood *et al*., 2020) (Figs 1, 2), scaling relationships between morphological variation and tissue properties (Price & Enquist, 2007) (Figs 3, 4), and how tissue geometry relates to function (Figs 5, 6). Our analyses support a departure from the *de facto* representation of spongy mesophyll as irregular or lacking order and establish that in many species the spongy mesophyll can be characterized instead by well‐conserved topological patterns. The honeycomb‐patterned spongy mesophyll obeys clear structural principles (Fig. 5) that emerge from allometric scaling properties linked to constraints imposed by cell (Figs 4, 6) and genome (Fig. S13) size. Investment in increased vein density and stomatal density enabled elevated rates of photosynthesis among the angiosperms (Brodribb & Feild, 2010; de Boer *et al*., 2012; Lehmeier *et al*., 2017; Muir, 2019), and these traits are apparently coordinated with the nonhoneycomb topology (Fig. S12). Our data suggest that cell size and cell packing density are critical for the development and specific structural configuration of a spongy mesophyll that has a high SA_mes_/*V*_mes_ (Fig. 6). This finding builds upon prior work linking cell size and CO_2_ diffusion supply within the leaf (Théroux‐Rancourt *et al*., 2021) and suggests that not only are cell size and cell packing density fundamentally limited by genome size (Simonin & Roddy, 2018; Roddy *et al*., 2020) (Fig. S11) but that the organization of the spongy mesophyll is similarly influenced. Shrinking the genome enables reductions in the sizes of stomatal guard cells, and mesophyll cells, collectively allowing for higher photosynthetic capacity by optimizing the hydraulic and diffusive pathways in the leaf (de Boer *et al*., 2012; Simonin & Roddy, 2018; Gago *et al*., 2019; Théroux‐Rancourt *et al*., 2021).

Our findings build on developmental work showing that hexagonal patterning can be found in numerous plant tissues and lineages, resulting from simple biophysical processes. For example, epithelial structures resolve into a honeycomb because this equalizes internal cell pressure within the population of developing and dividing cells (Lewis, 1928). Yet, the spongy mesophyll honeycombs enclose airspace voids instead of pressurized contents. In the 2D lattices of plant epithelia, planar space‐filling is optimized and the lattice is consequently more regular. By contrast, the hexagonally dominated, but less regular, 2D tessellation of the spongy mesophyll may be the result of lower (Barlow, 1974) cellular investment deep in the leaf, where photosynthetic cells are often light limited, whereas the vertical registration of the multiple cellular lattice layers, which position the IAS voids directly above the stomata, creates open channels for CO_2_ diffusion to the palisade, where light is abundant. Both temporal and spatial coordination have been observed between mesophyll airspace and epidermal cell differentiation during development (Lundgren *et al*., 2019). Such a topology suggests a minimization of construction costs in the spongy mesophyll while still meeting diffusive and biomechanical demands of the tissue. Future developmental studies could investigate the pathways for different spongy mesophyll phenotypes and the coordination of spongy mesophyll spatial organization with leaf traits such as stomatal patterning.

Honeycombs have been found widely in natural and engineered systems as multifunctional materials capable of balancing the demands of directional fluid transport, energy conversion, and structural support (Zhang *et al*., 2015). Honeycomblike structures have been observed within different types of plant tissues, such as the venation pattern of reticulate leaves (Price *et al*., 2012), where an approximately hexagonal topology has been argued to optimize transport efficiency of the vascular system (Fiorin *et al*., 2016). Leaves with higher vein density have an abundance of semirigid xylem conduits that provide mechanical support (Sack & Scoffoni, 2013). By contrast, the honeycomb structure itself is positioned between the upper and lower epidermises like in a sandwich beam (Gibson *et al*., 1988) and could produce a lightweight material that is elastic when loaded in the paradermal plane and stiff when loaded normal to the leaf surface, much like the manufactured honeycombs used in packing materials (Gibson & Ashby, 1999). A more complete understanding of leaf biomechanics now requires consideration of the spongy mesophyll phenotype. The hexagonal tessellation of the spongy mesophyll may, therefore, be the most efficient structure that satisfies multiple functional demands within a single tissue; that is, moving water over long distances outside the xylem, maintaining high diffusive conductance to CO_2_, exporting the products of photosynthesis, and serving as a self‐supporting structure when vein density is low. Hence, for plants with relatively large mesophyll cells, distantly spaced leaf veins, and moderate to low photosynthetic capacities, the emergent topological properties of the honeycomb structure likely provide a multifunctional strategy for economical resource allocation (Díaz *et al*., 2016).

## Author contributions

AMB conceived of the original research plan and jointly with CRB designed the methods, acquired, analyzed, and interpreted the data, and drafted and revised the manuscript. ABR acquired leaf microCT data and contributed to data analysis and manuscript revision. GT‐R acquired leaf microCT data and contributed to manuscript revision.

## Supporting information


**Fig. S1** Transverse and paradermal views of honeycomb mesophyll.
**Fig. S2** Measurement of arm cell dimensions.
**Fig. S3** Diagram showing minimum vein spacing measurements.
**Fig. S4** Nearest‐neighbor edge effect analysis.
**Fig. S5** Principal components analysis.
**Fig. S6** Cluster analysis.
**Fig. S7** Schematic of idealized cell geometries used in cell surface area and volume calculations.
**Fig. S8** Spongy mesophyll cell dimensions.
**Fig. S9** Comparison of PCA, cluster analysis, and observed phenotypes.
**Fig. S10** Spongy mesophyll structural outliers found in species with reticulate venation.
**Fig. S11** Structural variation in spongy mesophyll in leaves with parallel venation.
**Fig. S12** Partial dependence plots for random forest predictors.
**Fig. S13** Relationship between diploid C‐value genome size and mean arm cell diameter.
**Methods S1** Full details for IAS pore network analysis.
**Methods S2** Full details for nearest‐neighbor analysis.
**Methods S3** Full details for tessellation entropy, Lewis’ rule, and Aboav–Weaire law analysis.
**Methods S4** Full details for surface area and volume calculations of idealized cells.

**Table S1** 40‐species trait table.
**Table S2** Eigenvector scores of plant traits in three main PCA axes.
**Table S3** Confusion matrix for random forest classification of spongy mesophyll phenotype.
**Table S4** Phylogenetic nonindependence tests.
**Table S5** Pearson correlation matrix for 17 continuous leaf trait variables (*n* = 40 species).
**Table S6** Standardized major axis regressions.

**Video S1** MicroCT volume rendering of a coffee (*Coffea arabica*) leaf.

**Video S2** MicroCT volume rendering of a star anise (*Illicium floridanum*) leaf.

**Video S3** MicroCT volume rendering of a cork oak (*Quercus suber*) leaf.

**Video S4** MicroCT volume rendering of a cotton (*Gossypium hirsutum*) leaf.
Please note: Wiley Blackwell are not responsible for the content or functionality of any Supporting Information supplied by the authors. Any queries (other than missing material) should be directed to the *New Phytologist* Central Office.

## Data Availability

All data are available in the manuscript or deposited under the following doi on www.osf.io: 10.17605/OSF.IO/U59ZT.
