# Supplementary material for: Structural organization of the spongy mesophyll
Source: New Phytol. 2022 Feb 15;234(3):946–60. doi: 10.1111/nph.17971 (PMC9303971; doi:10.1111/nph.17971)
Supplement: Supplementary file 1 — Fig. S1 Transverse and paradermal views of honeycomb mesophyll. Fig. S2 Measurement of arm cell dimensions. Fig. S3 Diagram showing minimum vein spacing measurements. Fig. S4 Nearest‐neighbor edge effect analysis. Fig. S5 Principal components analysis. Fig. S6 Cluster analysis. Fig. S7 Schematic of idealized cell geometries used in cell surface area and volume calculations. Fig. S8 Spongy mesophyll cell dimensions. Fig. S9 Comparison of PCA, cluster analysis, and observed phenotypes. Fig. S10 Spongy mesophyll structural outliers found in species with reticulate venation. Fig. S11 Structural variation in spongy mesophyll in leaves with parallel venation. Fig. S12 Partial dependence plots for random forest predictors. Fig. S13 Relationship between diploid C‐value genome size and mean arm cell diameter. Methods S1 Full details for IAS pore network analysis. Methods S2 Full details for nearest‐neighbor analysis. Methods S3 Full details for tessellation entropy, Lewis’ rule, and Aboav–Weaire law analysis. Methods S4 Full details for surface area and volume calculations of idealized cells. [file NPH-234-946-s002.pdf]

New Phytologist Supporting Information

Article title: Structural organization of the spongy mesophyll

Authors: Aleca M. Borsuk, Adam B. Roddy, Guillaume Théroux-Rancourt, Craig R. Brodersen

Article acceptance date: 21 December 2021

The following Supporting Information is available for this article:

Fig. S1 Transverse and paradermal views of honeycomb mesophyll
Fig. S2 Measurement of arm cell dimensions
Fig. S3 Diagram showing minimum vein spacing measurements
Fig. S4 Nearest neighbors edge effect analysis
Fig. S5 Principal components analysis
Fig. S6 Cluster analysis
Fig. S7 Schematic of idealized cell geometries used in cell surface area and volume calculations
Fig. S8 Spongy mesophyll cell dimensions
Fig. S9 Comparison of PCA, cluster analysis, and observed phenotypes
Fig. S10 Spongy mesophyll structural outliers found in species with reticulate venation
Fig. S11 Structural variation in spongy mesophyll in leaves with parallel venation
Fig. S12 Partial dependence plots for random forest predictors
Fig. S13 Relationship between diploid C-value genome size and mean arm cell diameter
Table S1 (separate file). 40-species trait table.
Table S2 Eigenvector scores of plant traits in three main PCA axes
Table S3 Confusion matrix for random forest classification of spongy mesophyll phenotype
Table S4 Phylogenetic non-independence tests
Table S5 Pearson correlation matrix for 17 continuous leaf trait variables (n = 40 species)
Table S6 Standardized major axis regressionsMethods
Methods S1 Full details for IAS pore network analysis
Methods S2 Full details for nearest neighbors analysis
Methods S3 Full details for tesselation entropy, Lewis' rule, and Aboav-Weaire law analysis
Methods S4 Full details for surface area and volume calculations of idealized cells
Methods References
Video S1 (separate file). MicroCT volume rendering of a coffee leaf (*Coffea arabica*)
Video S2 (separate file). MicroCT volume rendering of a star anise leaf (*Illicium floridanum*)
Video S3 (separate file). MicroCT volume rendering of a cork oak leaf (*Quercus suber*)
Video S4 (separate file). MicroCT volume rendering of a cotton leaf (*Gossypium hirsutum*)

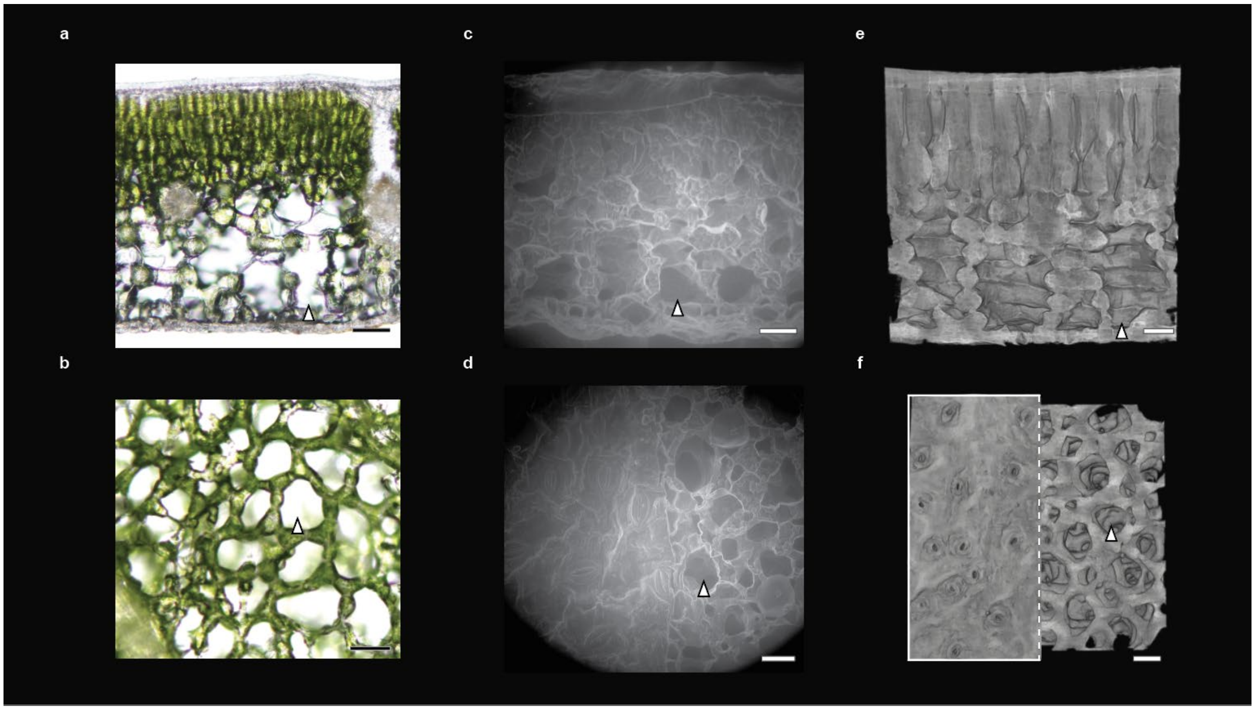

Fig. S1 | Transverse and paradermal views of honeycomb mesophyll. a-b, Brightfield microscopy (*Rhododendron* sp.). **c-d,** Environmental scanning electron microscopy (*Rhododendron* sp.). Paradermal view (**d**) shows both abaxial epidermis with stomata and a section of epidermis removed to show the spongy mesophyll. **e-f,** microCT (*Illicium anisatum*). Paradermal view (**f**) shows both abaxial epidermis with stomata and a section of epidermis removed to show the spongy mesophyll. White arrows indicate the positions of airspace voids. Bars = 50 μm.

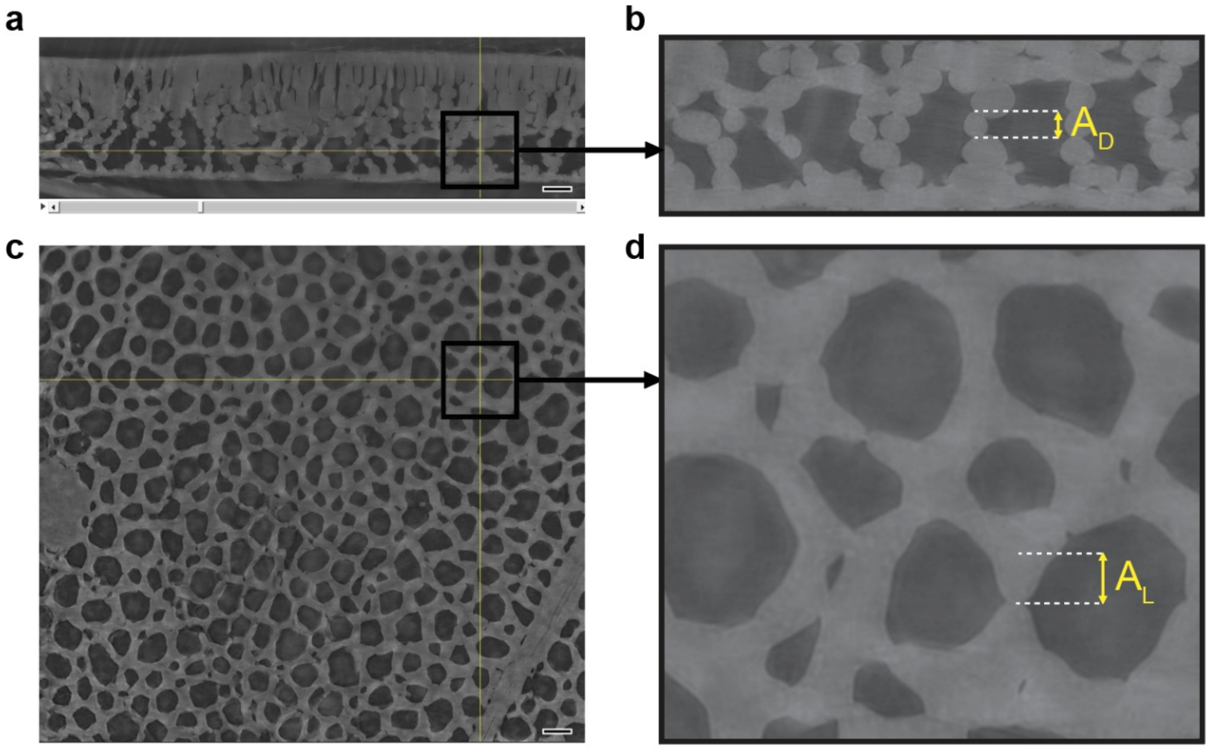

Fig. S2 | Measurement of arm cell dimensions. a, Transverse view of representative leaf with honeycomb spongy mesophyll. Yellow cross-hatch shows position of measured cell. **b,** Magnified view of **a** showing arm cell diameter (A_D) dimensions. **c,** Paradermal view of representative leaf with honeycomb spongy mesophyll. **d,** Magnified view of **c** showing arm cell length (A_L) dimensions. Bars = 50 µm.

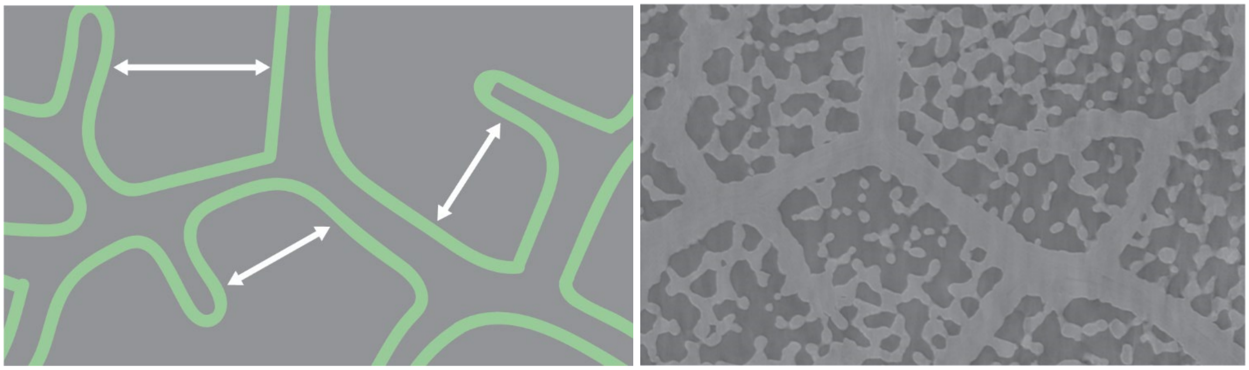

Fig. S3 | Diagram showing minimum vein spacing measurements. Measurements are made between the highest order vein and neighboring parallel vein (left panel) on microCT images (right panel).

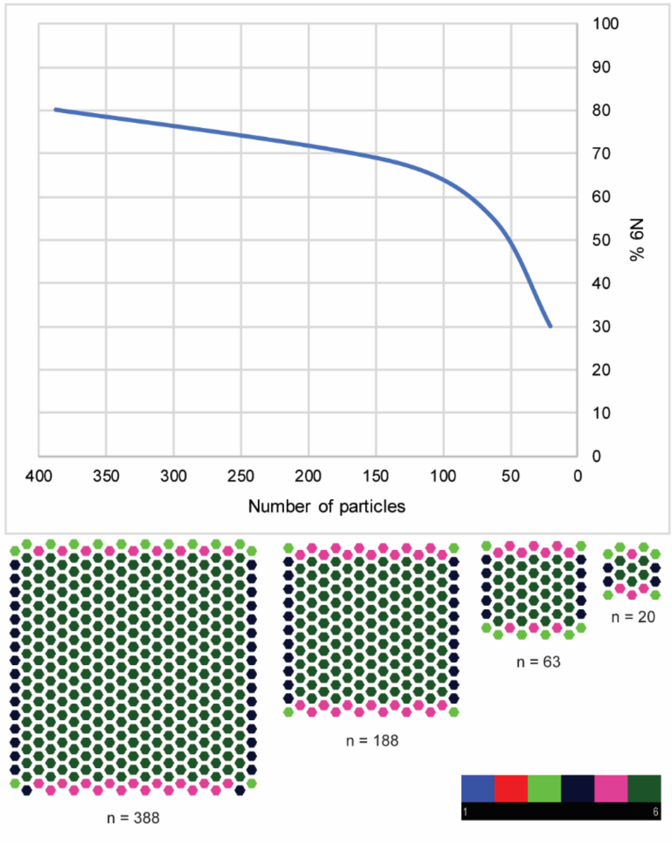

Fig. S4 | Nearest neighbors edge effect analysis. Influence of edge effects on model accuracy (percent of polygons with 6 neighbors, i.e. "% 6N") for a range of hexagonal aggregate sizes. The relationship between model accuracy due to edge effects and aggregate size was used to inform the lattice size (IAS pore counts) included in the nearest neighbor analysis of spongy mesophyll tissue (see Methods S2).

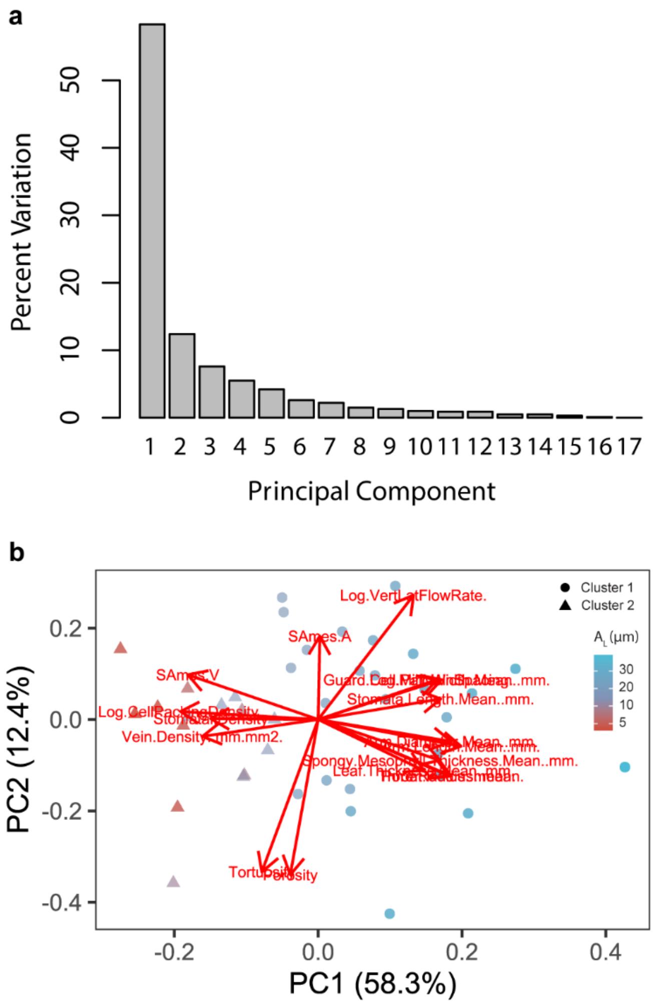

Fig. S5 | Principal components analysis. a, Screeplot showing the variation captured by each principal component. **b,** Biplot showing PCA scores for each species and loadings for each trait.

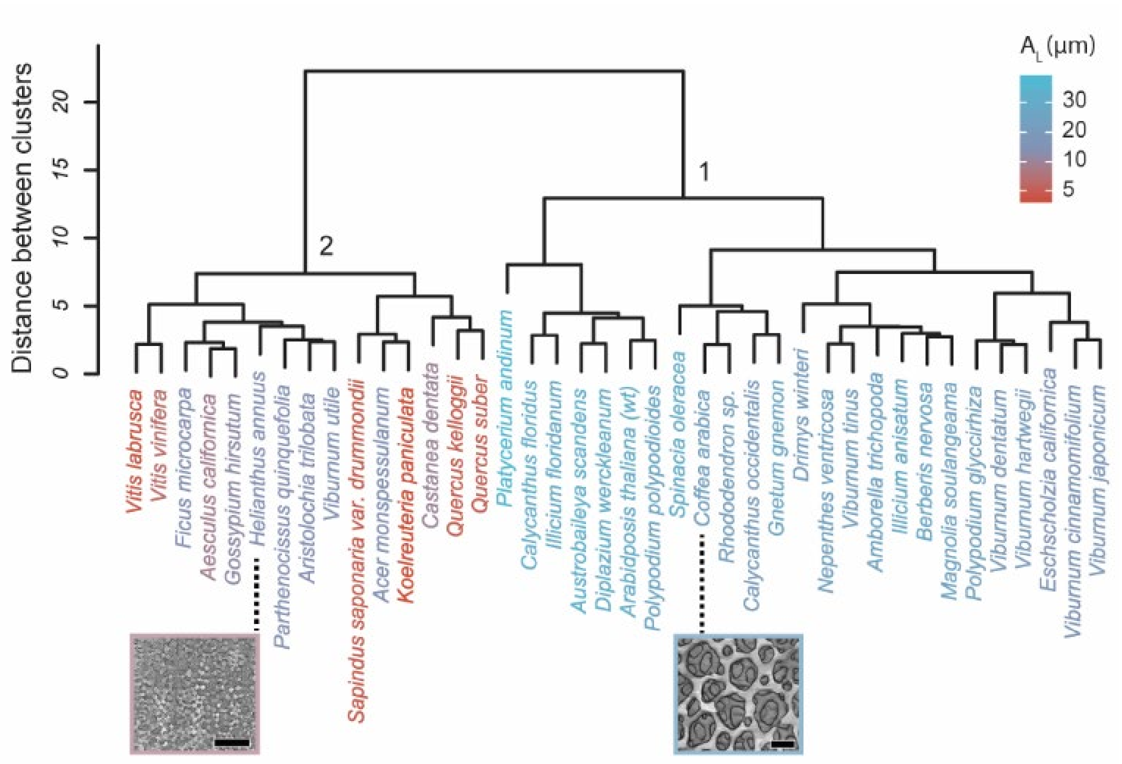

Fig. S6 | Cluster analysis. Dendrogram of cluster analysis relationships indicating the Euclidean distance between species in 17-dimensional space by Ward's agglomeration. Species clustered together are more similar. Two primary groupings indicated by numbers (Clusters 1 and 2). Species names are colored by mean cell arm length.

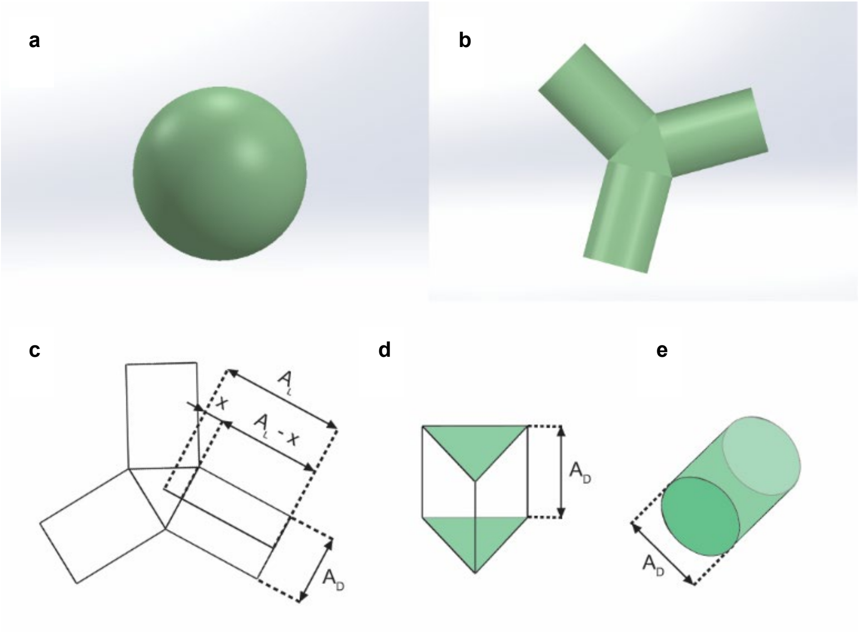

Fig. S7 | Schematic diagram of idealized cell geometries used in surface area and volume calculations for individual cells. a, Sphere. **b,** Triply-armed cell. **c,** Top view of triply-armed cell. **d,** Prismatic center of triply-armed cell. **e,** Cylindrical arm of triply-armed cell.

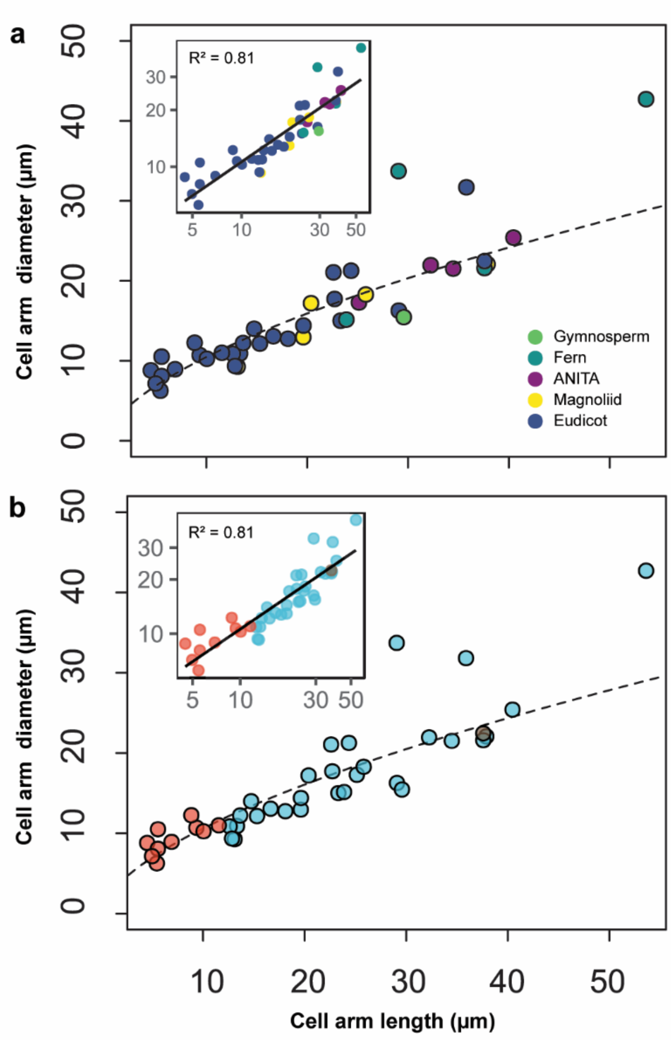

Fig. S8 | Spongy mesophyll cell dimensions. a-b, Relationship between spongy mesophyll mean cell arm diameter (A_D) and mean cell arm length (A_L). Power law regression shown by the black dashed line. Inset shows log-log transformed data and linear fit (solid line). Colors in (**a**) represent species major clade. Colors in (**b**) represent sample structural type (red = non-honeycomb, blue = honeycomb, brown = neither [*S. oleracea*]).

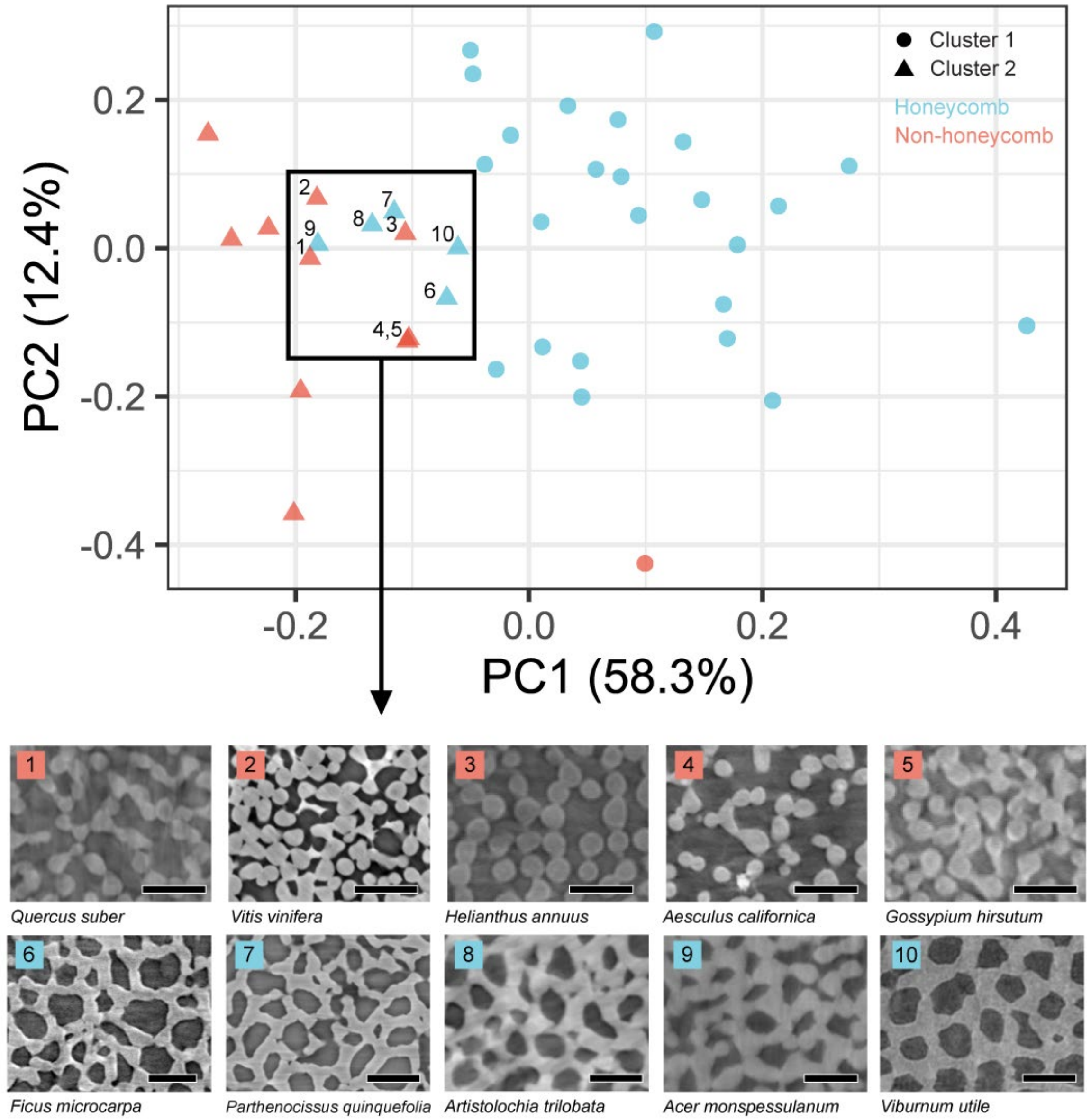

Fig. S9 | Comparison of PCA, cluster analysis, and observed phenotypes. PCA biplot showing the ordination of 40 species for 17 anatomical traits. Point shape indicates species representation in one of two primary groups identified through cluster analysis, using the same 17 anatomical traits. Point color indicates the presence of honeycomb (blue) or non-honeycomb (red) spongy mesophyll structural organization as identified through observation of lattice presence/absence in the paradermal plane and through analysis of the 3D IAS pore network geometry and associated flow rate directionality. Numbered points and corresponding images show examples of species with similar anatomical traits but different observed structural organizations. Points and images are numbered by increasing mean cell arm length (A_L; species 1, *Q. suber,* had the smallest A_L, effectively measured as cell radius for isodiametric cells). Blue triangles show species classified into Cluster 2 (short cell arm lengths; primarily non-honeycomb species) that were instead observed to have the honeycomb phenotype. Scale bars = 50 µm.

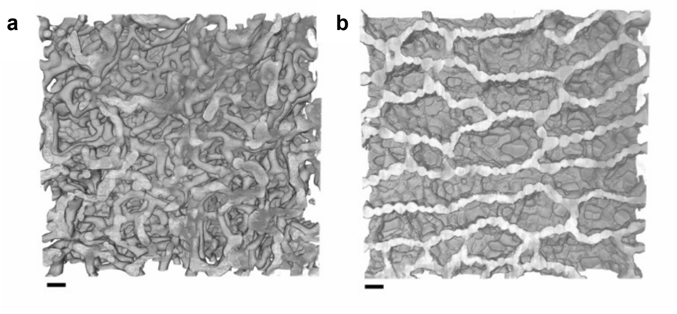

Fig. S10 | Spongy mesophyll structural outliers found in species with reticulate venation. a, Paradermal microCT images of the spongy mesophyll of spinach (*Spinacia oleracea*), with elongated cell arms forming large, convoluted and continuous airspaces. **b,** Paradermal microCT image of the spongy mesophyll of water lily (*Nuphar polysepala*), where multiple layers of the spongy mesophyll formed a lattice-like structure as found in species within the honeycomb class but arranged such that the IAS domains were elongated to form a highly porous tissue layer, presumably to increase buoyancy. Bars = 50 µm.

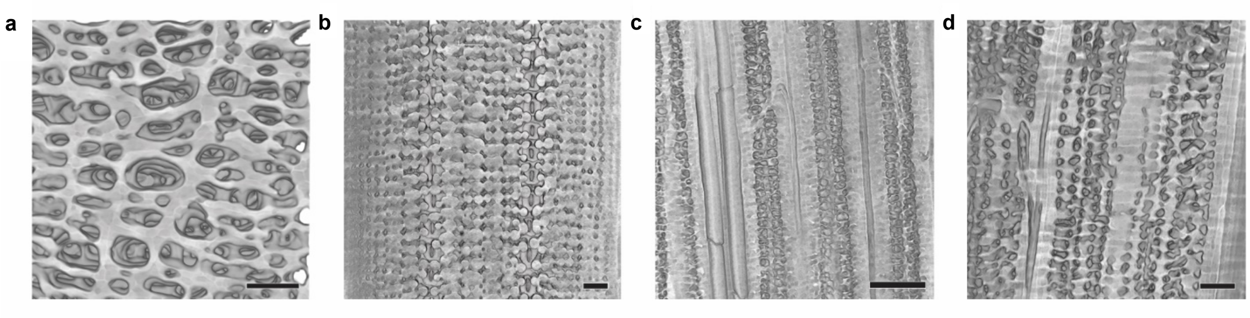

Fig. S11 | Structural variation in spongy mesophyll in leaves with parallel venation. **a,** Paradermal microCT image of *Cycas revoluta* with spongy mesophyll cells arranged in an orthogonal lattice. **b,** Paradermal microCT image of *Pinus monitcola* where the spongy mesophyll adjacent to the stomatal guard cells are arranged in an orthogonal lattice. **c,** Paradermal microCT image of the spongy mesophyll in rice (*Oryza sativa*) with parallel venation. **d,** Paradermal microCT image of the spongy mesophyll between the parallel veins of *Calamagrostis arundinacea*. Bars = 50 μm.

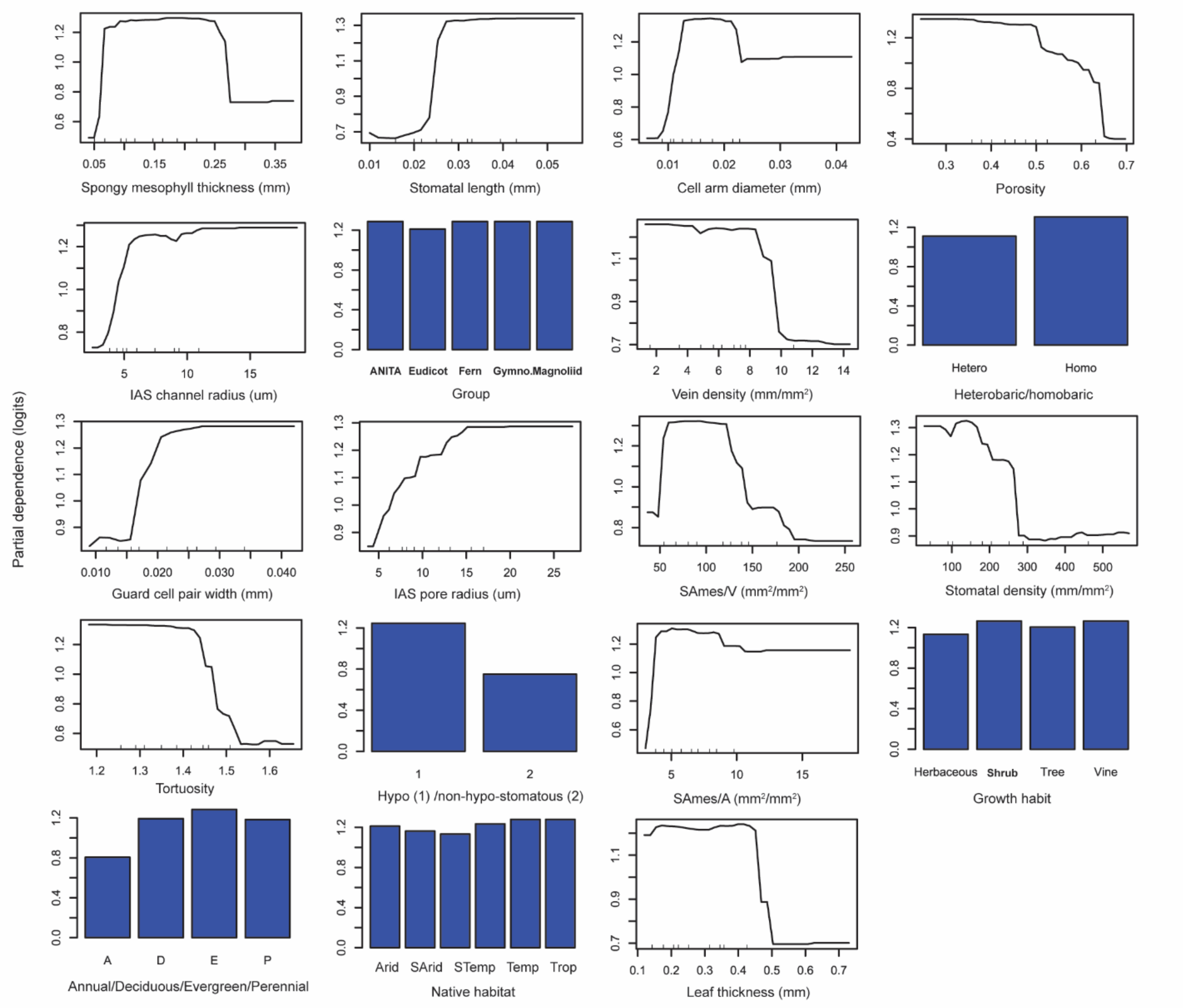

Fig. S12 | Partial dependence plots for random forest predictors. Graphical representation of marginal effect of anatomical, environmental, and taxonomic traits on the relative likelihood of classification as the non-honeycomb or honeycomb phenotype, reported as the partial dependence or natural logarithm of the odds (logit). Higher values indicate a higher relative probability of classification as the honeycomb phenotype. Partial dependence for the predictors with the four highest importance rankings shown in Fig. 4n-q.

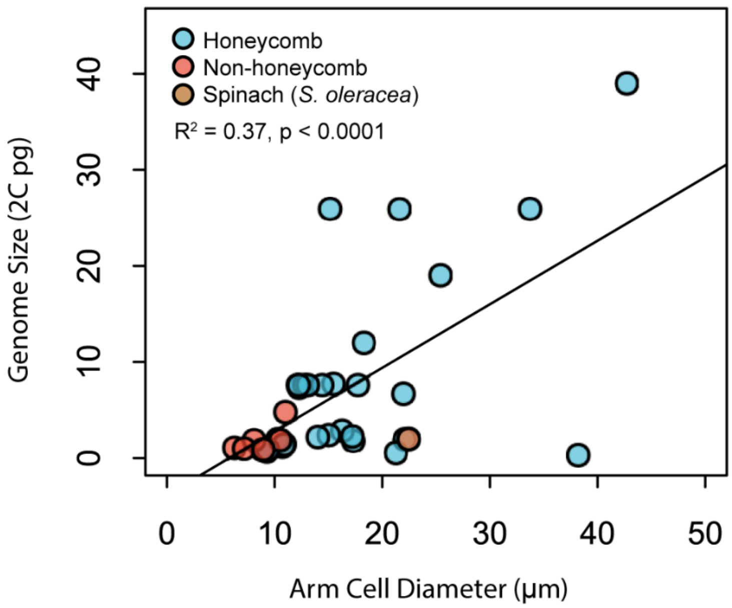

Fig. S13 | Relationship between diploid C-value genome size and mean arm cell diameter. Points colored by spongy mesophyll structural organization. Linear model shown by the black line ($R^2 = 0.37$, $F(1, 35) = 20.41$, $p < 0.0001$).

Trait	PC 1 (58.3%)	PC 2 (12.4%)	PC 3 (7.6%)
Leaf thickness	0.23	0.17	**0.46**
Spongy mesophyll thickness	0.26	0.14	**0.31**
Cell arm diameter	**0.29**	0.07	0.14
Cell arm length	**0.31**	0.09	-0.01
Minimum vein spacing	0.26	-0.13	-0.30
Vein density	-0.25	0.06	0.17
Stomata length	0.26	-0.07	-0.22
Guard cell pair width	0.25	-0.13	-0.17
Stomatal density	-0.23	0.00	0.27
Flow rate anisotropy	0.20	**-0.42**	0.03
Porosity	-0.06	**0.53**	-0.01
SA_{mes}/A	0.00	-0.28	**0.56**
SA_{mes}/V_{mes}	-0.28	-0.15	-0.06
Cell packing density	**-0.30**	-0.03	0.17
Tortuosity	-0.12	**0.51**	-0.20
IAS channel radius	**0.29**	0.19	0.13
IAS pore radius	0.28	0.19	0.09

Table S2 | Eigenvector scores of plant traits in three main PCA axes, obtained from a matrix of 17 traits × 40 species. The three highest eigenvector scores for each PCA axis are indicated in bold. Values in parentheses indicate variance accounted for by each axis.

	Honeycomb	Non-Honeycomb	Class Error
Honeycomb	29	0	0
Non-Honeycomb	1	10	0.09

Table S3 | Confusion matrix for random forest classification of spongy mesophyll phenotype.

		1	2	3	4	5	6	7	8	9	10	11	12	13	14	15	16
1	Arm diameter																
2	Arm length	**9.3, <0.001**															
3	Leaf thickness	**4.2, <0.001**	**2.8, <0.01**														
4	Spongy thickness	**5.2, <0.001**	**4.9, <0.001**	**11.1, <0.001**													
5	Vein density	**-2.1, <0.05**	**2.8, <0.01**	-0.2, 0.220	**-2.4, <0.05**												
6	Minimum vein spacing	**3.7, <0.001**	**6.3, <0.001**	-0.4, 0.725	1.1, 0.280	**-3.7, <0.001**											
7	Stomata length	**4.7, <0.001**	**4.4, <0.001**	1.4, 0.160	**2.5, <0.05**	**-3.2, <0.01**	**3.7, <0.001**										
8	Guard cell pair width	**5.6, <0.001**	**4.8, <0.001**	1.9, 0.061	**2.9, <0.01**	**-0.3.5, <0.01**	**4.0, <0.001**	**12.0, <0.001**									
9	Stomatal density	**-2.1, <0.05**	**-3.0, <0.01**	0.4, 0.712	-1.0, 0.342	**2.7, <0.05**	**-4.7, <0.001**	**-5.8, <0.001**	**-3.7, <0.001**								
10	SA_{mes}/V_{mes}	**-13.5, <0.001**	**-10, <0.001**	**-5.3, <0.001**	**-7.1, <0.001**	**2.6, <0.05**	**-3.1, <0.01**	**-4.1, <0.001**	**-4.3, <0.001**	1.9, 0.072							
11	SA_{mes}/A	1.1, 0.286	-0.57, 0.572	1.9, 0.061	1.7, 0.097	0.1, 0.945	-1.13, 0.268	0.7, 0.519	1.4, 0.158	0.8, 0.439	-0.3, 0.733						
12	Porosity	-0.25, 0.803	0.43, 0.665	-0.2, 0.858	0.1, 0.937	-0.3, 0.775	0.1, 0.984	1.0, 0.329	0.7, 0.464	-1.1, 0.268	-0.7, 0.495	-1.2, 0.224					
13	Cell packing density	**-7.0, <0.001**	**-14.1, <0.001**	-2.0, 0.052	**-3.5, <0.01**	**4.0, <0.001**	**-7.0, <0.001**	**-4.5, <0.001**	**-5.3, <0.001**	**2.6, <0.05**	**7.5, <0.001**	0.6, 0.549	-0.9, 0.399				
14	Tortuosity	-0.67, 0.507	-0.79, 0.431	-1.4, 0.172	-1.1, 0.283	0.2, 0.832	0.0, 0.992	-0.2, 0.814	-0.5, 0.643	-1.8, 0.070	0.8, 0.423	-1.2, 0.219	**3.6, <0.001**	0.1, 0.943			
15	IAS channel radius	**9.3, <0.001**	**9.7, <0.001**	**4.5, <0.001**	**5.3, <0.001**	**-2.9, <0.01**	**3.0, <0.01**	**3.6, <0.001**	**4.1, <0.001**	-1.6, 0.117	**-16.0, <0.001**	0.1, 0.958	1.3, 0.200	**-8.0, <0.001**	-0.5, 0.612		
16	IAS pore radius	**7.7, <0.001**	**7.8, <0.001**	**5.0, <0.001**	**5.1, <0.001**	**-2.3, <0.05**	**2.4, <0.05**	**3.6, <0.01**	**3.9, <0.001**	-1.3, 0.204	**-11.8, <0.001**	-0.2, 0.860	1.1, 0.265	**-6.6, <0.001**	-0.8, 0.417	**22.1, <0.001**	
17	Vertical:lateral flow rate	**2.1, <0.05**	**3.0, <0.01**	0.8, 0.436	1.5, 0.154	**-2.2, <0.05**	**3.1, <0.01**	0.9, 0.391	1.5, 0.131	-0.5, 0.610	-1.8, 0.082	0.6, 0.528	**-3.5, <0.01**	**-2.4, <0.05**	**-5.0, <0.001**	1.4, 0.169	1.1, 0.284

Degrees of freedom = 37; t-values, and p-values reported in each cell.
Statistically significant, statistically non-significant

Table S4 | Degrees of freedom, t-values, and p-values for pairwise generalized linear models taking into account phylogenetic non-independence between data points for 40 species.

		1	2	3	4	5	6	7	8	9	10	11	12	13	14	15	16
1	Arm diameter																
2	Arm length	**0.89**[a]															
3	Leaf thickness	**0.75**[a]	**0.69**[a]														
4	Spongy thickness	**0.76**[a]	**0.83**[a]	**0.87**[a]													
5	Vein density	**-0.60**[a]	**-0.71**[a]	**-0.46**[b]	**-0.61**[a]												
6	Minimum vein spacing	**0.70**[a]	**0.82**[a]	**0.37**[c]	**0.56**[a]	**-0.70**[a]											
7	Stomata length	**0.67**[a]	**0.78**[a]	**0.39**[b]	**0.54**[a]	**-0.68**[a]	**0.73**[a]										
8	Guard cell pair width	**0.71**[a]	**0.70**[a]	**0.42**[b]	**0.50**[a]	**-0.62**[a]	**0.73**[a]	**0.85**[a]									
9	Stomatal density	**-0.60**[a]	**-0.66**[a]	**-0.41**[b]	**-0.55**[a]	**0.77**[a]	**-0.66**[a]	**-0.71**[a]	**-0.57**[a]								
10	SA_{mes}/V_{mes}	**-0.80**[a]	**-0.87**[a]	**-0.67**[a]	**-0.75**[a]	**0.59**[a]	**-0.66**[a]	**-0.67**[a]	**-0.65**[a]	**0.56**[a]							
11	SA_{mes}/A	0.04	-0.06	0.11	0.01	-0.07	-0.13	0.05	0.03	0	0.05						
12	Porosity	-0.17	-0.1	0	-0.05	0.19	**-0.29**[c]	-0.1	-0.18	0.13	-0.05	-0.15					
13	Cell packing density	**-0.78**[a]	**-0.92**[a]	**-0.54**[a]	**-0.69**[a]	**0.72**[a]	**-0.86**[a]	**-0.77**[a]	**-0.74**[a]	**0.66**[a]	**0.87**[a]	0.15	0.15				
14	Tortuosity	-0.27	-0.28	-0.21	-0.21	0.2	**-0.37**[c]	**-0.35**[c]	**-0.45**[b]	0.08	0.23	-0.28	**0.53**[a]	0.29			
15	IAS channel radius	**0.87**[a]	**0.93**[a]	**0.73**[a]	**0.79**[a]	**-0.61**[a]	**0.74**[a]	**0.66**[a]	**0.61**[a]	**-0.54**[a]	**-0.85**[a]	-0.04	0	**-0.83**[a]	-0.19		
16	IAS pore radius	**0.84**[a]	**0.90**[a]	**0.71**[a]	**0.74**[a]	**-0.58**[a]	**0.74**[a]	**0.67**[a]	**0.60**[a]	**-0.52**[a]	**-0.85**[a]	-0.11	-0.02	**-0.82**[a]	-0.22	**0.98**[a]	
17	Vertical:lateral flow rate	**0.46**[b]	**0.57**[a]	**0.33**[c]	**0.43**[b]	**-0.53**[a]	**0.63**[a]	**0.52**[a]	**0.55**[a]	**-0.44**[b]	**-0.61**[a]	0.2	**-0.50**[a]	**-0.61**[a]	**-0.71**[a]	**0.42**[b]	**0.38**[c]

[a]$p < 0.001$
[b]$0.001 \le p < 0.01$
[c]$0.01 \le p < 0.05$
Statistically significant, statistically non-significant

Table S5 | Pearson correlation matrix for 17 continuous leaf trait variables (n = 40 species).

Traits	Slope	Elevation	R^2	p
Cell arm diameter ~ Cell arm length	0.6576211 (0.5697227, 0.7590806)	0.3533032 (0.2331697, 0.4734367)	0.807802	3.5012e-15
Cell packing density ~ Cell arm length	-1.782334 (-1.940287, -1.637240)	-0.32425451 (-0.59500219, -0.05350684)	0.9329849	2.22e-16
Flow rate anisotropy ~ Cell arm length	3.296124 (2.558926, 4.245701)	7.007994 (5.497058, 8.518931)	0.3929272	1.5071e-05
Minimum vein spacing ~ Cell arm length	1.1995632 (0.9896222, 1.4540415)	1.3210542 (0.9056773, 1.7364311)	0.6525377	2.9753e-10
SA_{mes}/V_{mes} ~ Cell arm length	-0.7161596 (-0.8208904, -0.6247906)	2.900018 (2.775625, 3.024412)	0.8261927	5.1249e-16

Table S6 | Standardized major axis regressions for allometric scaling relationships (n = 40 species). Estimates for slope and elevation are reported, followed by lower and upper limits in parentheses.

Methods S1.

Pore network analysis. To sample a consistent number of IAS pores among species, tissue was cropped to a pore count of approximately 4 by 4 IAS pores in the lateral plane. The vertical height of the cropped sample was maximized to the thickness of spongy mesophyll tissue present in the leaf. The image stack was imported into the Avizo XPoreNetworkModeling Extension and the airspace and tissue were thresholded into binary objects using the Auto Thresholding module ("Low" setting). To measure the fraction of connected pores, or the connectivity of the IAS network, unconnected pores were removed using the Axis Connectivity module oriented along the lateral (z) axis. IAS connectivity was measured by running the Volume Fraction module on each of the total airspace and interconnected airspace objects. To model the IAS as a set of connected and labeled pores, the Separate Objects module was used on the interconnected IAS object. neighborhood This was optimized for arbitrary (non-spherical) pore shapes using the Skeleton -Aggressive setting, marker extent of 1, and using the connected object output type with a repeatable algorithm mode. The resulting object was a set of IAS domains with network properties obtained using the Generate Pore Network Model module. The Generate Properties function was used to approximate vapor phase flow through the network using boundary values of 40 Pa and 25 Pa as input and output pressure, respectively, and a fluid viscosity of 1.837 x 10^{-5} Pa s. Flow rate was calculated where the mass conservation for each node (pore) was described as:

$$\sum_{i \to j} q_{ij} = 0 \tag{1}$$

with the summation performed on each node *j* connected to node *i* and q_{ij} representing the flow rate between node *i* and *j*. Assuming laminar flow conditions, the relation between pressure drop and flow rate was modeled as linear:

$$q_{ij} = g_{ij} \, (P_i - P_j) \tag{2}$$

with g_{ij} representing the conductance of the channel between nodes *i* and *j*. Using cylinders to represent network channels of radius r_{ij} and length l_{ij}, the conductance was approximated by:

$$g_{ij} = \frac{\pi}{8\mu} \frac{r_{ij}^4}{l_{ij}} \tag{3}$$

Imposing a pressure difference across the network gave a linear system of equations which was solved numerically, with (1)(2) leading to the matrix equations: G * P = S where G, the matrix of conductance, was a symmetrical matrix of dimension N * N where N was the number of nodes in the network, P was a vector of size N corresponding to the pressure in each node, and S was a vector of size N constrained by the pressure boundary conditions. The total flow rate *Q* was then computed as:

$$Q = \sum(P_i - P_j)g_{ij} \quad (4)$$

on each pair of nodes *i,j* intersecting an arbitrary cross section of surface A. Transport was measured in the lateral (z) and again in the vertical (y) direction by changing the Direction setting for each simulation.

Methods S2.

Nearest neighbors. Paradermal microCT slices were made within the spongy and cropped to exclude veins. Edge pixels were smoothed using the Gaussian Blur filter (0-10 pixel radius of decay). The "watershed irregular features" function for the ImageJ plugin Biovoxxel (Brocher, 2015) was used to repair gaps introduced in the tissue structure by thresholding, and by cross-referencing the binary image with the grayscale image stack. Images were despeckled using the Median filter. The "nearest neighbors" function for the ImageJ plugin Biovoxxel was used to classify pore polygon class using the UEP Voronoi analysis method with pixel size parameters 0-infinity (mm^2). Parameters were set to exclude edge particles from the computation and from the image output. An edge effect was observed where pores at the edges of an image had fewer neighbors compared to pores in the image center. Therefore, the influence of edge effects on the nearest neighbor distribution were analyzed (Fig. S4).

Methods S3.

Tesselation entropy, Lewis' rule, and Aboav-Weaire law. Tessellation entropy (*S*) describes the degree of order in a system by its polygon class distribution (Pietsch *et al.*, 2009), where (*n*) is the number of nearest neighbors for each polygon and *P*(*n*) is the probability of finding *n* nearest neighbors in the system. This was calculated using the nearest neighbors counts for each species according to:

$$S = -\sum_n P(n) \ln[P(n)] \quad (8)$$

with *S* = 0 for a perfectly regular structure. Polygons with fewer than four or greater than nine sides were excluded. Mean tessellation entropy and standard deviation was then calculated (n = 29).

The Aboav-Weaire Law (Weaire, 1974) maintains that any face, i.e. IAS pore, with a lower than average number of edges ($n < 6$) will introduce a corresponding face with a higher number of edges, so that any five-sided face will have a seven-sided partner within the tessellation, frequently as a neighbor. This is given as:

$$\bar{m} = 5 + \frac{6}{n} \quad (9)$$

where n is the number of edges corresponding to a particular face and $\bar{m}$ is the average number of edges of its n neighbors.

Similarly, Lewis's Law (Lewis, 1928) relates topological patterns of size dispersion in honeycombs where the area of a given face varies linearly with its number of edges as:

$$\frac{A(n)}{A(\bar{n})} = \frac{n - n_o}{\bar{n} - n_o} \qquad (10)$$

where $A(n)$ is the area of a cell with n sides, $A(\bar{n})$ is that of a cell with the average number of sides, and n_o is a constant (Lewis finds $n_o = 2$). On average, this gives larger faces more neighbors.

To calculate the Lewis ratio ($A(n)/A(\bar{n})$) and Aboav-Weaire edge average for each polygon class ($\bar{m}$), the paradermal binary watershed images prepared in the previous step were opened in FIJI and converted to a Voronoi polygon image. Binary Voronoi images were then opened in the Icy image processing software package (de Chaumont *et al.*, 2012) with a modified (Vetter *et al.*, 2019) EpiTools plugin (Heller *et al.*, 2016). The CellGraph module was used first to create a map of the different IAS polygons while excluding edge domains. Using the EpiTools plugin for Icy, we identified all IAS polygons captured in the images, determined the IAS area inscribed by the cell wall boundaries, determined the number of edges of the IAS domains, the number of neighboring IAS polygons, and the area and number of sides of those neighbors. In some species, the largest polygon classes have a higher than anticipated mean area; this is likely because occasionally two or more IAS polygons were combined during image thresholding due to local variation in image contrast or because cells were out of plane.

Methods S4.

Surface area and volume calculations of idealized isodiametric and triply-lobed cells. Volume of a triply-lobed cell ($V_{arm\ cell}$) was first calculated according to:

$$V_{arm\ cell} = \frac{\sqrt{3}}{4}A_D^3 + 3[\pi\left(\frac{A_D}{2}\right)^2(A_L - \frac{1}{2}\sqrt{\left(\frac{A_D}{2}\right)^2 + (A_D)^2})] \qquad (11)$$

with cell diameter A_D and cell arm length A_L. Surface area of the arm cell ($SA_{arm\ cell}$) included the upper and lower faces of the triangular prism and the side and (one) base of each cylinder:

$$SA_{arm\ cell} = \frac{\sqrt{3}}{2}A_D^2 + 3[(\pi A_D)(A_L - \frac{1}{2}\sqrt{\left(\frac{A_D}{2}\right)^2 + (A_D)^2}) + \pi\left(\frac{A_D}{2}\right)^2] \qquad (12)$$

Volume was considered as conserved between the arm cell and isodiametric cell:

$$V_{isodiametric\ cell} = V_{arm\ cell} \quad (13)$$

and surface area of the isodiametric cell ($SA_{isodiametric\ cell}$) was then calculated as:

$$SA_{isodiametric\ cell} = 4\pi r^2 \quad (14)$$

with the isodiametric cell radius (r) given by:

$$r = \sqrt[3]{\frac{3V_{isodiametric\ cell}}{4\pi}} \quad (15)$$

Surface area to volume ratios were then calculated for each cell type using the measured A_D and A_L values.

Methods References

Brocher J. **2015**. The BioVoxxel Image Processing and Analaysis Toolbox. In European BioImage Analysis Symposium 2015 Jan 5.

de Chaumont F, Dallongeville S, Chenouard N, Hervé N, Pop S, Provoost T, Meas-Yedid V, Pankajakshan P, Lecomte T, Le Montagner Y, *et al.* 2012. Icy: an open bioimage informatics platform for extended reproducible research. *Nature Methods* **9**: 690–696.

Heller D, Hoppe A, Restrepo S, Gatti L, Tournier AL, Tapon N, Basler K, Mao Y. **2016**. EpiTools: An Open-Source Image Analysis Toolkit for Quantifying Epithelial Growth Dynamics. *Developmental Cell* **36**: 103–116.

Lewis FT. **1928**. The correlation between cell division and the shapes and sizes of prismatic cells in the epidermis of cucumis. *The Anatomical Record* **38**: 341–376.

Pietsch T, Gindy N, Fahmi A. **2009**. Nano- and micro-sized honeycomb patterns through hierarchical self-assembly of metal-loaded diblock copolymer vesicles. *Soft Matter* **5**: 2188–2197.

Vetter R, Kokic M, Gómez H, Hodel L, Gjeta B, Iannini A, Villa-Fombuena G, Casares F, Iber D. **2019**. Aboave-Weaire's law in epithelia results from an angle constraint in contiguous polygonal lattices. *bioRxiv*: 591461.

Weaire D. **1974**. Some remarks on the arrangement of grains in a polycrystal. *Metallography* **7**: 157–160.

Video S1 (separate file). MicroCT volume rendering of a coffee leaf (*Coffea arabica*) with a paradermal bisection, opening to reveal the honeycomb structure of the spongy mesophyll. Tissue dimensions are approximately 700 μm across the horizontal edge. Scale varies with perspective.

Video S2 (separate file). MicroCT volume rendering of a star anise leaf (*Illicium floridanum*) with a paradermal bisection, opening to reveal the honeycomb structure of the spongy mesophyll. Tissue dimensions are approximately 700 μm across the horizontal edge. Scale varies with perspective.

Video S3 (separate file). MicroCT volume rendering of a cork oak leaf (*Quercus suber*) with a paradermal bisection, opening to reveal the non-honeycomb structure of the spongy mesophyll. Tissue dimensions are approximately 700 μm across the horizontal edge. Scale varies with perspective.

Video S4 (separate file). MicroCT volume rendering of a cotton leaf (*Gossypium hirsutum*) with a paradermal bisection, opening to reveal the non-honeycomb structure of the spongy mesophyll. Tissue dimensions are approximately 700 μm across the horizontal edge. Scale varies with perspective.
